# Beating Tunnel Vision: Near-Surface Velocity-Map Imaging

**DOI:** 10.1021/acs.jpca.5c08379

**Published:** 2026-02-17

**Authors:** Nitish Pal, Preeti M. Mishra, Paul D. Lane, Matthew L. Costen, Kenneth G. McKendrick, Stuart J. Greaves

**Affiliations:** † Institute of Chemical Sciences, Heriot-Watt University, Edinburgh EH14 4AS, U.K.; ‡ Department of Physics, Government Women’s College Keonjhar, Mandua 758001 Odisha, India

## Abstract

We demonstrate a new methodology for applying velocity-map imaging (VMI) to the study of gas-surface dynamics that allows direct imaging of the scattering plane. By bringing the surface near to the ionization laser inside the VMI electrodes, we demonstrate a route to overcoming “tunnel vision”, a term used to describe the limited angular acceptance of measurements taken at too great distance from where the gas-surface interaction takes place. Using a molecular beam of NO molecules colliding with a surface of highly oriented pyrolytic graphite as an exemplar system, we show the correlation between the laser-surface distance and the accessible scattering angular range. We demonstrate the capabilities of near-surface VMI (NS-VMI) through the analysis of speed and angular distributions derived from the images.

## Graphical abstract

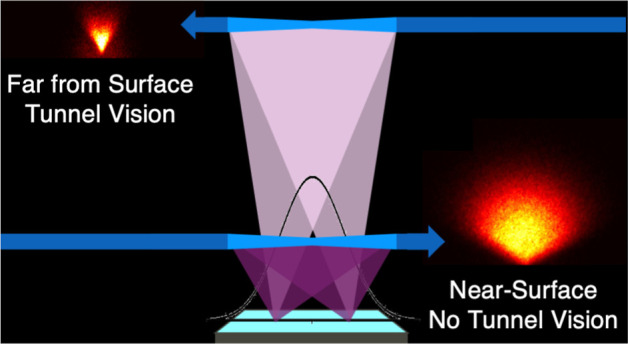

## 1 Introduction

Scattering of gas-phase molecules at a solid or liquid interface is important in many chemical processes; e.g., respiration, catalysis, combustion, and aerosol chemistry. Given the importance of such processes, gaining a clear understanding of the chemical dynamics and mechanisms that occur is highly desirable. Experimental techniques have been developed to examine different aspects of interfacial processes with increasing levels of detail, especially for gas–liquid processes which, because of their technical challenges, have until recently received less attention.

To control the initial conditions of gas–liquid surface collisions, molecular-beam (MB) scattering has been used by a significant number of research groups^1–12^ to produce defined speeds, incident angles, and cold internal states of the gas molecules impinging on the surface. The liquid surfaces in these experiments were either produced by the wetted wheel technique^11,13–15^ or, less commonly, by liquid microjets and leaves^16–20^ of low-vapor-pressure liquids or salty-water solutions.^21–23^ MB scattering has also been employed widely in the study of gas–solid surface interactions, a popular area of study whose comprehensive review is beyond the scope of this publication; however an example pertinent to the current work, due to the shared detection method, is metal surfaces being studied for their application to catalysis.^24–26^ Another example of relevance is the study of highly oriented pyrolytic graphite (HOPG) as a proxy for spacecraft shielding.^27,28^ Due to the flatness of the graphitic layers in HOPG, it has been shown to give very narrow specular angle scattering distributions for high incident speeds.^29^ In particular, the scattering of NO from HOPG is a well-studied, popular system that has been shown to produce highly directional surface scattering.^30–32^ These narrow scattering distributions also make it an ideal test surface for new surface-scattering-analysis techniques.

In scattering experiments, speed and angular information play a crucial role in revealing the mechanisms that govern the formation of scattered products. Parameters such as energy exchange, sticking coefficient, surface structure, and the nature of the collision (inelastic or reactive) can be extracted from speed and angular distributions.^2,3,11,12,23^

Detection of surface-scattered products has been achieved by a variety of methods. The first experimental detection approaches used mass spectrometers (MS) to record the scattered species at defined angles relative to the surface normal (defined as 0°).^1,2,5,12,33^ Using choppers for the in-going molecular beam as well as for the scattered molecules before they entered the MS allowed good measurements of the speeds of the scattered products with well-defined angles. However, this approach could only measure certain specific scattering angles, e.g., back scattering along the incidence angle was excluded due to geometric constraints. Furthermore, the universal detection methods used in mass spectrometry do not allow the measurement of the internal energy of the products. Without internal-energy measurements, the full dynamics and energy partitioning of surface collisions cannot be determined.

To determine the internal energy of the scattered products, our own group and that of Nesbitt have used laser-spectrometric techniques including direct absorption to detect molecules such as CN, CO_2_, and HF scattering from liquid surfaces of squalane or perfluoropolyether (PFPE).^7,10,15,34^ Both groups and, in early work, the group of McCaffery have also used laser-induced fluorescence (LIF) to record the inelastic scattering of I_2_, OH, and NO, as well as the OH products of the reactive scattering of O­(^3^P), from a variety of surfaces including squalane, squalene, PFPE, and ionic liquids.^9,15,35–45^ The fundamental advantage of state-specific spectroscopic schemes is that they provide detailed information on the population of different ro-vibrational states after collision. Early spectroscopic experiments probed all scattered products in a particular spectroscopically selected quantum state, and so could not provide the velocity of the products, though some later experiments used Doppler-resolution or pointwise measurements relative to a surface impact point to gain limited scattering-angle and speed information.^7,10,11,34,46–48^

More detailed information can be measured by using LIF imaging^38,49,50^ employing a planar laser sheet to probe the scattered products enabling simultaneous acquisition of both internal energy and velocity information for OH radicals scattered from a series of liquids. Measurement of velocity and internal-state distributions enables analysis of correlations between molecular speed and angular distribution, for well-defined precollision velocities and internal states. While LIF imaging is an excellent technique for providing the necessary information to interpret the dynamics of some gas-surface collisions, it has its limitations; it is only applicable to scattered species that have accessible electronic states that can fluoresce, and the extraction of speed information involves the analysis of a temporal sequence of spatially resolved images, for which careful calibration is required.^38,49,50^

The gas-phase scattering community, which has the same desire to record both the internal state and velocity of collision (or photolytic) products, has seen a near-ubiquitous adoption of the velocity-map imaging (VMI) technique.^51,52^ When combined with a state-specific ionization scheme, VMI is able to provide all the information needed,^53^ in principle, from a single-point measurement at a single delay. VMI typically uses a series of annular electrodes (ion-optics) that have been designed carefully to generate an electric field that maps all ions with the same velocity onto the same point on a position-sensitive detector irrespective of their starting position. Note that the VMI ion-optic that sits furthest from the detector and with the highest voltage difference is called the repeller, and the next electrode is typically named the extractor.

Applying the advantages of VMI to gas-surface scattering (surface-VMI) is more technically demanding than for gas-phase scattering due to the requirement to accomodate a surface with which the gas-phase molecules collide. Nevertheless, it has been attempted by several research groups, typically using one of two different approaches; either by placing the surface of interest on the repeller of the ion-optics, or by placing the surface outside the annulus of the ion-optics.

Surface-VMI experimental geometries that place the surface on the repeller, pioneered by Wodtke^54^ and co-workers and adopted by Nesbitt and co-workers,^15,26,55,56^ face several restrictions on the scattering systems that can be studied. The surface used must not affect the electric field produced by the repeller; this typically limits the surfaces to metals, self-assembled monolayers, or near-monolayer thin coatings. Geometric constraints are also generally imposed; these severely limit the available incident angles of the impinging gas as any elements of the apparatus needed to generate the molecular beam must not interfere with the electric fields or block the time-of-flight axis of the ions generated, while ensuring that the incident molecular beam can pass between/through the ion-optics. This meant that normal-incidence experiments were effectively impossible. A further constraint in such experiments is the inability to directly image the 2D scattering plane, which is defined by the (non-normal) incident molecular beam(s) and the surface normal (see Figure 1a). Since the surface is mounted on the repeller, which is parallel to the plane of the detector, the scattering plane is oriented perpendicular to the imaging plane. Consequently, a basic VMI image only provides direct information on out-of-plane velocities. It is possible, in principle, to recover the velocity information in the scattering plane through use of a sufficiently fast time-sliced detector, along with velocity calibration in the time-of-flight direction. However, it is technically challenging to achieve the necessary time resolution and requires rigorous data analysis and detailed modeling to deconvolute the scattered velocities as a function of scattering angle.

**Figure 1:**
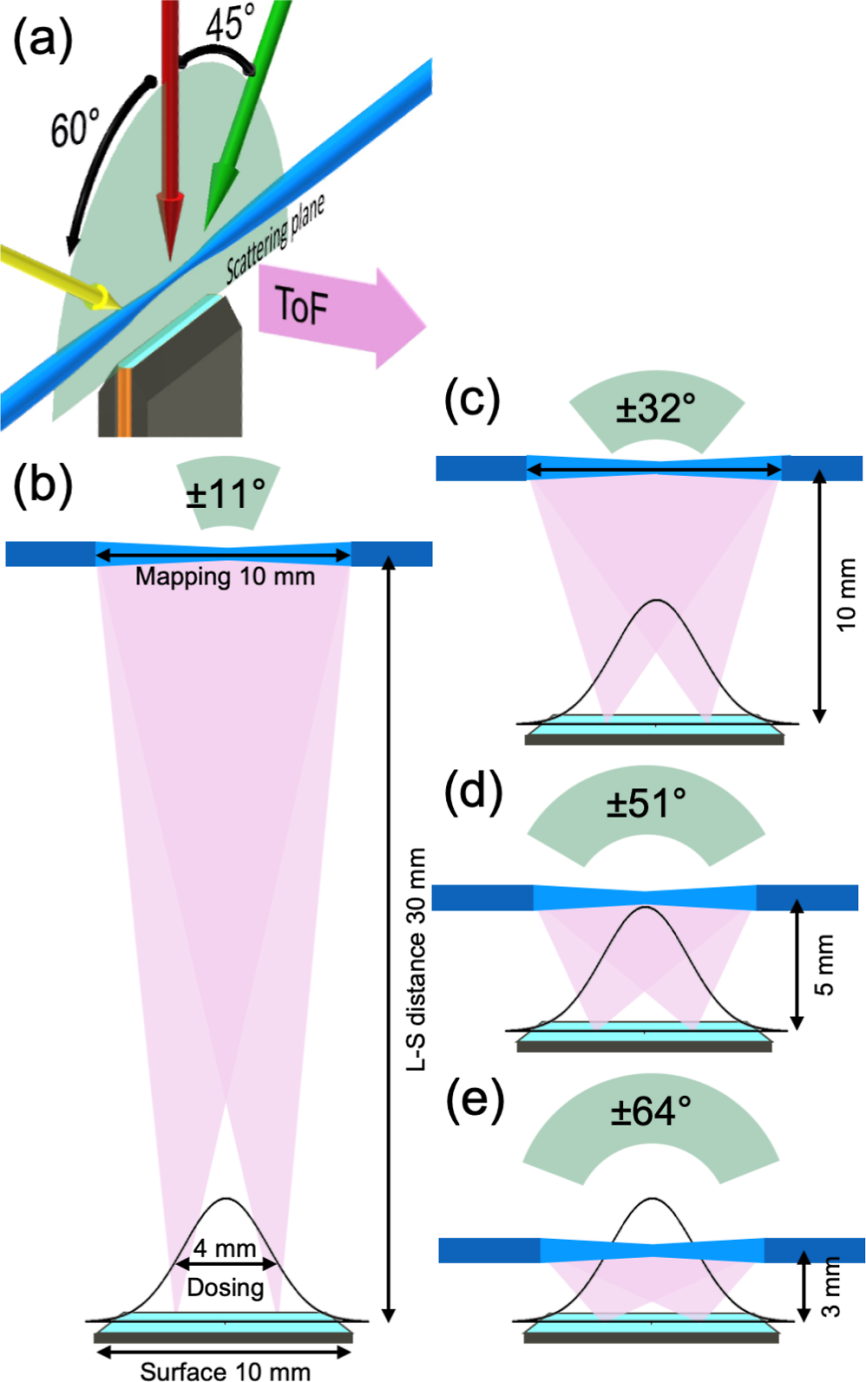
(a) Scattering plane schematic; in a surface-scattering experiment, the scattering plane (translucent green semicircle) is defined as the plane containing the surface normal and the axes of the molecular beams (yellow, red, and green arrows) incident on the surface. (b) The range of detectable scattering angles in a surface-scattering experiment with laser-based detection is defined and constrained by several parameters: the size of the surface (turquoise, 10 mm), the area of the surface dosed by the incident molecular beam (Gaussian profile, fwhm = 4 mm), the smallest size of the ionization or velocity-mapping regions (light blue, 10 mm), and the laser-surface distance (L-S distance 30 mm). The angular profile caused by these parameters has a range of ±11° about the surface normal (dosing-weighted intensity that encompassed 95% of the possible scattering intensity). (c) When all other parameters are kept constant and the L-S distance is reduced to 10 mm, the angular profile increases to ±32°. (d) For an L-S distance of 5 mm, the angular profile is ±51°. (e) For an L-S distance of 3 mm, the angular profile is ±64°. The dimensions indicated in panels (c–e) are specific to the experimental setup described in this paper; similar constraints will be observed for other experiments employing VMI for surface-scattering measurements, see the text for details.

Placing the surface outside the ion-optics, as used, e.g., in the groups of Wodtke^57^ and Koehler,^58^ avoids many of the problems of the surface-on-repeller geometries. In this configuration, the surface plane lies perpendicular to the imaging detector and hence the velocities that are mapped are those in the scattering plane. Moreover, the molecular beam can now be directed at the surface at any incident angle, using the unrestricted opening between the repeller and extractor plates. However, because of the necessary distance between the surface and the ionization laser due to the radius of the VMI electrodes, a new limitation is imposed; we dub this here “tunnel vision”.^59^ Because of the distance between the surface and the ionization/velocity-mapping region, only those molecules scattering into a narrow range of angles about the surface normal can be detected (the work of Greenwood and Koehler reports their angular acceptance as ±14°).^58^ It is as if the detection volume is effectively looking at the surface down a long tunnel. Tunnel vision is a consequence of all the factors that restrict the range of surface-scattering angles that can be detected in this form of the VMI experiment, as illustrated in Figure 1b. The length of the ionization region (typically the focused Rayleigh range of the ionization-laser beam) is an important factor; if it is too short, the range of detected scattering angles will be limited, too long and ions may be created outside the velocity-mapping region of the ion-optics. It is the smaller of the Rayleigh range or length of the mapping region that limits the diameter of the “tunnel”. Equally, the laser-surface (L-S) distance is crucial in determining the detectable angular range because it is equivalent to the length of the tunnel. It is the ratio of these two distances which defines the range of detectable scattering angles, provided the dosed area of the surface is sufficiently large. In practice, the finite size of the dosed area on the surface may also need to be considered, either because of the finite length (in the direction parallel to the scattering plane) of the surface itself or more likely because of the fraction of that length that can be dosed by a molecular beam. A well-collimated beam may not uniformly cover the surface and the consequences of the uneven dosing must be taken into account.

The consequences for the detectable angular range are illustrated for representative values (as used in this paper) of these parameters in Figure 1c–e, where the L-S distance is allowed to reduce while other factors are fixed. The parameters used in these calculations are derived from known experimental geometrical factors and VMI simulations, as described in the Supporting Information Section SI-1. Figure 1b uses an L-S distance of 30 mm, similar to that used in previous experiments,^57,58^ with the remaining parameters the same as this work to illustrate the limited angular range detectable when far from the surface. In comparison at the furthest distance used in this work (L-S = 10 mm), an angular range of ±32° about the surface normal has been modeled to be observable (encompassing the central 95% of the possible weighted scattering intensity). When the other parameters are kept constant and the L-S distance is reduced to 5 mm, the angular profile increases to ±51°; this further increases to ±64° for L-S = 3 mm.

It is instructive to consider how some hypothetical cases would affect the detectable angular range: if the length of the ionization region was reduced (and became shorter than the mapping region), e.g., by the need for tighter focusing for a multiphoton process, this would reduce the detectable angular range. Likewise, if a smaller surface was used (e.g., 1 × 1 mm), this would also reduce the detectable range. Both of these effects could be mitigated by reducing the L-S distance, and a larger dosed area on the surface would also mitigate the reduction in probe-region length. In the limit that the ionization volume is very close to the dosed area of the surface, the other experimental factors are not important; any correlation between probe volume and detectable scattering angle is removed, and all angles become detectable.

To circumvent the tunnel-vision problem, we present here an evolution of our previous work^60^ that allows the surface of interest to be placed *inside* the velocity-mapping electric fields at different L-S distances near to the laser ionization region. By using this “Near-Surface Velocity-Map Imaging” (NS-VMI), we aim to overcome the limitations of previous surface-VMI techniques and provide more complete state-selected measurements of the whole 2D scattering plane. We will describe the technical requirements for implementing NS-VMI, examine its velocity-mapping capabilities with surfaces inside the electric fields before demonstrating the technique’s capabilities with measurements and analysis of the scattering of NO molecules that collide at normal incidence with a HOPG surface.

## 2 Experimental Methods

The NS-VMI experiment is described in this section, with specifics given for the apparatus, the VMI detection technique, and our modifications to it that enable the surface to be placed near to the laser-ionization region. We will also explain the mechanism for introducing the surface to the experiment and adjusting it within the ion-optics and the effect of this on reducing tunnel vision. Due to the complex nature of the experimental setup, more details can be found in the Supporting Information Section SI-2.

### 2.1 NS-VMI Geometries

The key features of the geometries of the NS-VMI experiment are shown in Figure 2. The main components were based on those of a typical VMI experiment, with a set of annular electrodes coaxial with a time-of-flight axis (*Y*-axis, horizontal in the lab frame) that leads to an imaging detector. The surface is introduced along the *Z*-axis (vertical in the lab frame) from below the ion-optics, allowing it to pass between the electrodes and approach the path of the ionization-laser beam. The laser propagates along the *X*-axis (horizontal in the lab frame), intersecting the central axis of the ion-optics, and parallel to both the plane of the imaging detector and the surface. For the gas-surface-scattering experiments, NO molecules were prepared in a MB that propagated along the *Z*-axis toward the surface from above the ion-optics. The apparatus has been designed to also allow MBs to propagate in the *XZ* plane at incident angles of 45° and 60° relative to the surface normal (see Supporting Information Figure S2), although only scattering from the normal incidence MB is presented here. The scattering plane is the *XZ* plane that includes the MBs, the surface normal (*Z*), and the laser beam (*X*), as illustrated in Figure 1.

**Figure 2:**
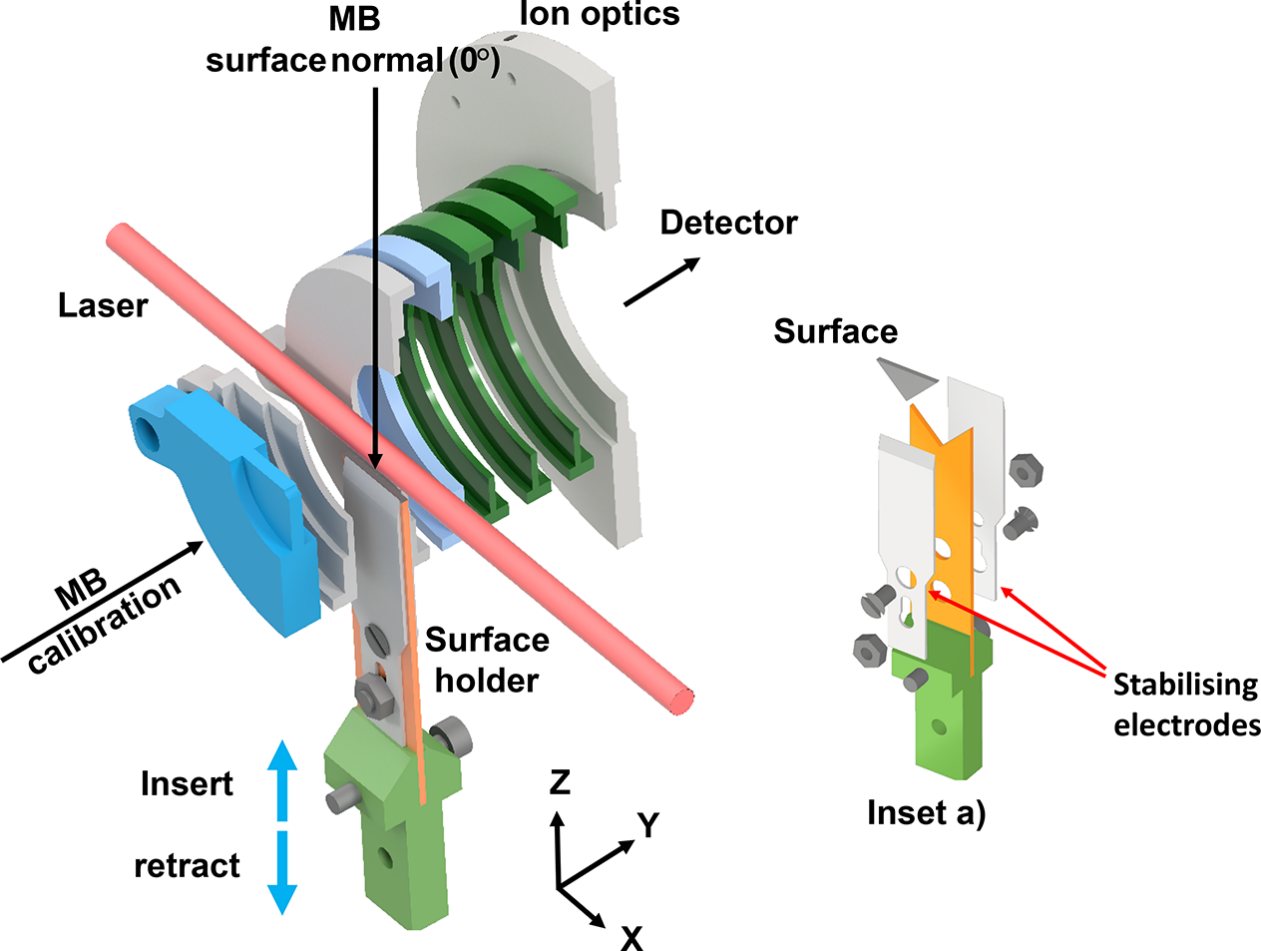
NS-VMI experimental schematic showing the geometry of the key features of the experiment. A subset of the ion-optics is shown in a cut-away form around the surface holder. The surface holder is introduced from below the ion-optics, and its position relative to the laser beam can be adjusted. The two molecular-beam geometries, indicated by labeled arrows, correspond to the calibration and scattering geometries used for the experiments described in the text. The inset (a) shows an exploded diagram of the surface, its holder, and its stabilizing electrodes. See the text for more details.

To demonstrate how the velocity-mapping is affected by the presence of the surface inside the electrodes, photodissociation experiments were conducted in the calibration geometry where the MB was propagated along the *Y*-axis, coaxial with the ion-optics. This calibration-geometry minimized the spread of molecular velocities parallel to the detector plane (i.e., in the *XZ* plane, see Figure 2) prior to photodissociation, a factor that would distort product velocities if the molecular beam was introduced along the *Z*-axis (as used in the scattering experiments). The calibration-geometry molecular beam was generated using a pulsed solenoid valve (Parker General valve, series 9, 1 mm diameter orifice). It was skimmed by a 1.01 mm diameter skimmer (Beam Dynamics, model 1) that was mounted with its tip ∼34 mm from the laser axis. Further details of the experimental apparatus and the calibration geometry can be found in the Supporting Information (Section SI-2).

### 2.2 Gas-Surface-Scattering Methodology

The MB used for surface scattering experiments was generated using another Parker General valve with an 0.5 mm orifice. The MB was skimmed by a 0.5 mm diameter skimmer (Beam Dynamics, model 1) the base of which was mounted 72.3 mm from the laser axis. The valve and skimmer were mounted from the top of the scattering chamber (see Supporting Information Figure S2b) with the molecular-beam assembly being positioned above the baffle that divides the scattering chamber into source and ionization regions. The baffle is formed of a horizontal metal plate with a semicylindrical center section that encloses the ion-optics. A 1 mm wide slit on the curved surface of the baffle further collimates the molecular beam before it enters the ion-optics. Both the source and ionization regions are evacuated by the same DN250CF turbomolecular pump (Pfeiffer, ATP 2300M, 1700–2050 l/s for He) that keeps the source and ionization regions at pressures of 3.1 × 10^–6^ and 1.3 × 10^–6^ mbar, respectively, during operation. This design minimizes the path length to the pump for molecules which rebound from the chamber walls, both above and below the baffle.^61^

The surface used in the experiments presented here was HOPG with surface dimensions along the *X-* and *Y*-axes of 10 × 1 mm (Agar scientific (AGF1800–1–1), see Supporting Information Section 2.2 for characterization details). The HOPG surface was cleaned using the sticky tape method and then mounted on top of a polyether ether ketone (PEEK) holder of the same cross section which extends below the surface to a region outside of the ion-optics (see the orange part in the inset exploded diagram in Figure 2). Kapton film was used to electrically isolate the surface from the stabilizing electrodes either side of the surface that will be discussed in more detail in Section 2.3 below.

The HOPG surface is located within the ion-optics below the baffle such that the slit-collimated MB (traveling along the *Z*-axis) can collide with its upper face (*XY* plane) at normal incidence. The surface (and attendant equipment, see inset in Figure 2) is positioned using an XYZ-translator (LewVac M-XYZ-12–406–63CF) that provides micrometer precision adjustment along the *Y* and *X*-axes and can retract the surface vertically (along the *Z*-axis) from the ionization region into a load lock that sits below the main chamber. The control over the *Z* position allows experiments to be run at a series of laser-surface (L-S) distances, as shown in Figure 1. The L-S distance is measured by raising the surface until it occludes half of the laser beam, then lowering it by the required amount. This procedure is carried out for every L-S distance change to eliminate the effect of backlash from the long *Z*-axis translator.

The size of the dosed area on the surface is a key parameter for surface-scattering, as illustrated in Figure 1 and associated text. This was determined by using spatial-map imaging (SMI) (see Section 2.3 below). In essence, this simply involves applying a different set of voltages to the same ion-optics. The measurement showed that the MB had a full-width at half-maximum of ∼4 mm, meaning that 99% of the MB hit the surface, and 95% of the dosing is in the central 7 mm of the 10 mm surface.

### 2.3 VMI

The ion-optics used here were a modified version of those used in previous gas-phase scattering experiments,^62,63^ whose design was based on a combination of those of Townsend et al.^64^ and Lin et al.^65^ They are comprised of 20 annular electrodes to create three electric field regions with a low electric-field in the ionization region and softer focusing fields through the ion-optics stack to improve velocity resolution. To achieve best performance, a balance between competing experimental considerations is required: (1) the ionization region in which effective velocity mapping is achieved should be as long as possible, in the *X*-direction, to maximize the range of detectable surface-scattering angles; (2) the molecular-beam flux onto the surface should be maximized; (3) there should be sufficient space in the *Y*-direction between the electrodes either side of the scattering region to allow both the introduction of the surface and efficient pumping of the scattering region; (4) the design should minimize field distortion when a surface is introduced; (5) the velocity-mapping resolution should be high-enough to allow expected scattering features to be measured faithfully. Considerations (2) and (3) call for small-diameter ion-optics to allow the MB source to be closer to the ionization region, and for well-separated electrodes on either side of the region for efficient pumping. These considerations were balanced with consideration (1) which calls for the opposite. Consideration (5) informs the balance between (1) and (2)/(3) as the typical features seen in surface-scattering experiments are significantly broader in terms of speed (e.g., features with a full width half maximum of several hundreds of meters per second)^57^ than those found in gas-scattering experiments, allowing some deterioration in mapping quality to be acceptable.

To address consideration (4), the breadth (i.e., the dimension along the time-of flight *Y* axis) of the dielectric surface is kept to a minimum while still being large enough to enable straightforward manufacture of the surface. A broader surface increases the length of perturbed field through which an ion has to travel. Crucially, adjustable-voltage stabilizing electrodes were placed on either side of the surface to compensate for the field perturbation caused by the introduction of a dielectric into the VMI fields. The stabilizing electrodes consisted of two scalpel blades (A.C.M. 18), selected because their sharp top edge (optically determined to be no greater than 40 μm thick) had the dual benefit of minimizing any molecular scattering from the blades and reducing electric field perturbations.

The ion-optics could be operated in both VMI mode and SMI mode, as noted above (see Supporting Information Sections 3.1 and 3.2 for details). The ion-optics design also included a 16-plate deflector in the field-free region that could be used to fine-tune the position of the ion cloud on the detector. This was included as previous experiments showed the possibility of the surface assembly shifting the ion cloud vertically onto the detector; however, it was not required for any of the measurements reported here. Further details of the ion-optics including dimensions, optimized voltages, and electric fields can be found in the Supporting Information Section SI-3.

Ions were created by photoionization using the doubled output of a pulsed dye laser (Sirah Cobra-Stretch) pumped by a 20 Hz Nd/YAG laser (Continuum Surelite, SL I-20). The ∼2 mm diameter laser beam was focused into the chamber; details of lenses, typical laser powers, and the ionization schemes used are provided in relevant sections below. After acceleration by the ion-optics, the ions were recorded by a combination of a 40 mm diameter in-vacuum position-sensitive detector (Photek VID240, GM-MCP-2, P46 phosphor) and a Basler CMOS (a2A1920–160umBAS) camera. The detector is run in a pulsed mode to select just the mass-to-charge of interest unless otherwise stated. The timings of the detector, laser, and the solenoid valve were set by a delay generator (Quantum composer 9520 series). The experiment was run by custom LabView data acquisition and control software developed in our laboratory.

## 3 Results and Discussion

### 3.1 Calibration of VMI

The desire to minimize the L-S distance, as explained above, was tensioned against the perturbation of the velocity-mapping fields caused by a dielectric surface positioned directly below the ionization region, even with stabilizing electrodes on either side. To quantify the effect of this perturbation, photodissociation experiments were conducted with the surface at a series of L-S distances.

These experiments used the one-color photodissociation probing the 3dπ­(^3^Σ_g_^+^(*v* = 2, *N* = 2) Rydberg state and subsequent ionization (via multiple processes)^66^ of oxygen atoms from O_2_ molecules; this approach has long been used to characterize VMI experiments and demonstrate their imaging quality.^26,51,52,62,65,66^ All measurements in this subsection used the calibration geometry (described above and in the Supporting Information) with the MB directed along the *Y*-axis toward the detector, coaxial with the ion-optics. The MB was generated with neat O_2_ (BOC 99.9995%, 3 bar backing pressure) and was intersected by a laser beam of wavelength 224.999 nm (Coumarin 450 laser dye, ∼1 mJ pulse energy) that was focused into the ionization region using a lens (Thorlabs, UV-fused silica plano-convex 300 mm). The linear polarization of the laser light was vertical (along the *Z*-axis) in the lab frame. DC-slicing^64^ of the Newton sphere of O^+^ ions with a detector gate width of 25 ns was used to record the images without the surface present. Using these images, the velocity calibration of the detector was established to be 5.4 ms^–1^/pixel for O^+^ ions (*m*/*z* = 16), as shown in Supporting Information Figure S8.

The O_2_-photodissociation measurements were repeated with the HOPG surface introduced into the ion-optics at three different L-S distances of 3, 5, and 10 mm. Typical images for these L-S distances, along with a no-surface image, are shown in the central column of Figure 3. Some blurring of the rings as the L-S distance is reduced can be seen but distinct rings are visible in all images.

**Figure 3:**
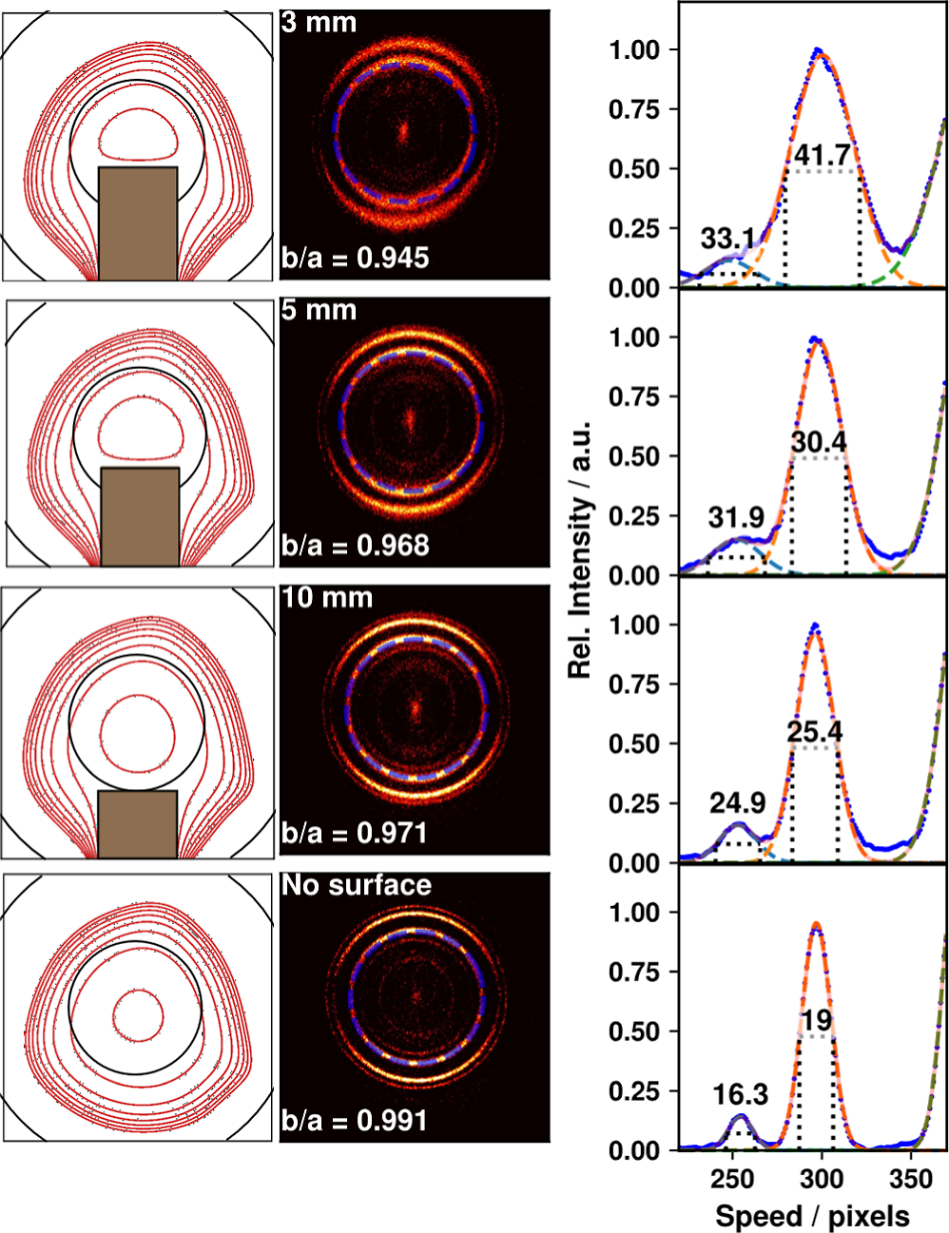
VMI electric fields, calibration images, and speed distributions for different L-S distances. The first column shows SIMION simulations of the electric fields (1 V contours as red lines) in the scattering plane (*XZ*) for the ion-optics with no surface (bottom row), and with L-S distances of 10, 5, and 3 mm, in the third to first rows, respectively. The black circle in the center of each contour plot represents the location of the aperture in the closest electrode, and the black arcs in the corners represent the outside edge of the same electrode. The brown rectangle shows the location of the stabilizing electrode. The second column shows velocity-mapped images of O_2_ photodissociation at 224.999 nm at each distance. The white text overlaid at the bottom of each image indicates the *b*/*a* ratio of the upper part of the blue dashed line shown in the images. The third column shows in blue the speed distribution of the upper portion of the ring highlighted by the blue dashed line in the second column. The speed distribution was fitted (red dashed line) with a Gaussian distribution for each ring, and the fwhm of the fits is displayed on each graph. See the text for details.

Introducing the surface breaks the cylindrical symmetry of the fields created by the ion-optics, as demonstrated in the first column of Figure 3 which shows the electric field contours (red lines are 1 V contours between 950 and 957 V) in the ionization region as simulated using SIMION software (version 8.1, Scientific Instrument Services, Inc.).^67^ The lack of closely spaced contours along the laser ionization region (*X*-axis) above the 10 mm surface (the field gradient here was only 0.1 V mm^–1^) demonstrated the small scale of the perturbation caused by the introduction of the surface into the ion-optics. It can also be seen that the field curvature in the upper part of the images in this column remains the same irrespective of the presence of the surface; trajectories of ions traveling through this region (i.e., those that are scattered from the surface) will be minimally influenced by the presence of the surface.

The circularity of the O_2_-photodissociation images was used to measure the effect of the L-S distance on the cylindrical symmetry of the focusing field. The circularity was examined by fitting an ellipse to the upper (i.e., with velocities away from the surface) part of the sliced Newton rings in the images (shown in the central column of Figure 3 with a dashed blue circle) and extracting the ratio of major and minor axes, denoted by *a* and *b*, respectively. Only the upper part of the image (positive *Z*) was used for this analysis (and also the analysis of speeds in the third column) because it is this part of the image that will contain any signal in surface-scattering experiments; ions with trajectories that take them closer to the surface (negative *Z* velocities) are more likely to suffer from distortion due to the asymmetric electric fields in that direction as shown in the first column. A perfect circle corresponds to zero eccentricity (i.e., *b*/*a* = 1). Despite breaking the cylindrical symmetry of the ion-optics by introducing the surface, the figure shows only a small effect: from *b*/*a* = 0.991 for no surface, reducing to 0.945 at L-S distance of 3 mm. A more subtle effect of introducing the surface is a small shift (1–10 pixels) in the position of the image center. For these photodissociation images, this can be determined easily by calculating the center (zero-velocity) pixel as part of the image analysis procedure.

The third column in Figure 3 shows the radially integrated speed distribution around all angles of the upper (i.e., with velocities away from the surface) part of the image. The most pronounced effect of introducing a surface into the ion-optics is the decrease in speed resolution presented in this column. Each image’s speed distribution was fitted with a Gaussian distribution for each ring, and the fwhm of the fits in pixels is displayed in Figure 3. The width of the red-fitted peak is ∼100 ms^–1^ without any surface present, this increases progressively to ∼225 ms^–1^ at a L-S distance of 3 mm. When compared with the broad speed distributions generally expected for surface-scattering processes (fwhm ∼ 500 ms^–1^),^57^ this result is within acceptable limits.

The results shown in Figure 3 for an HOPG surface are typical of those recorded for different surface materials. Supporting Information Figure S9 shows O_2_-photodissociation images recorded for four different surface materials: HOPG, polytetrafluoroethylene (PTFE), polyether ether ketone (PEEK), and mica. No surface-charging effects were observed for any of the materials studied. There are no major differences in the effects of these four surfaces on the quality of the images, with any observed differences being within the day-to-day variability of measurements. Greater speed blurring and increased distortion of the cylindrically symmetry were observed with a 2 mm breadth (along the *Y*-axis) PEEK surface. The results presented in the remainder of this paper were all taken using a 1 mm breadth surface. The consistent performance across this range of materials, with dielectric constants ranging from 2 to ∼20,^68^ demonstrates that our stabilizing electrodes mitigate the influence of widely varying surfaces on the velocity-mapping fields.

In summary, these O_2_-photodissociation experiments demonstrate that VMI is still possible even with a surface placed near the ionization region. Minimal angular distortion is caused by the surface, and the speed blur observed is within acceptable limits.

### 3.2 Scattering of NO From HOPG

#### 3.2.1 Experimental Considerations and Procedures

For the surface-scattering measurements, the experiment was changed from calibration to scattering geometry (as described in the experimental section 2.2 and Figure 2), with a molecular beam of NO colliders propagating along the *Z*-axis from above. For this configuration, we have also confirmed the detector calibration using the photodissociation of NO_2_ which creates NO molecules that were subsequently ionized by the same laser pulse (∼226 nm, Coumarin 450 laser dye) via a [1 + 1] REMPI scheme probing the NO­(A^2^Σ^+^ – *X* ^2^Π) (0,0) band.^69^ This provided a velocity-to-pixel calibration of 3.98 ms^–1^/pixel for NO^+^ (*m*/*z* = 30), confirming the calibration obtained by mass-converting the value derived from the O_2_ photodissociation experiments. This also demonstrated the VMI quality for species with similar velocities to those that were expected in the scattering experiments in the presence of a surface; further details can be found in the Supporting Information, see Section SI-3.4.

During surface-scattering, NO^+^ ions can be created at any point along the laser path accessible to NO molecules. In the present experiments, this is an advantage as it allows the maximum possible range of surface-scattering angles to be detected without being restricted by the Rayleigh length of a focused laser beam.

The present NS-VMI surface-scattering experiments we now describe did not need to use dc-slicing because the experimental geometry, a small diameter laser above a relatively narrow surface, ensures that only scattered molecules strongly confined to the *XZ* plane can propagate from the surface into the probe-laser volume to be ionized and thus detected. In effect, the experimental geometry allows NS-VMI to “optically slice”^70^ only the scattering plane. The narrow out-of-plane angular ranges are shown in Table 1 below and in Supporting Information Table S1.

**Table 1:** Detectable Out of Scattering Plane Angular Ranges of the Central 95% and 68% of the Cumulative Intensity of the Surface-Dosing-Weighted Geometric Histograms^a^

L-S distance / mm	95% out of plane	68% out of plane
3	±9.7°	±6.4°
5	±5.9°	±3.8°
10	±3.0°	±1.9°

a Values are shown for the different laser surface distances used, see Supporting Information Section 1 for details.

To scatter NO from the surface, a molecular beam with a pulse width of 200 μs (fwhm), was generated using a mixture of 1% NO (BOC > 99.9%) in He (BOC > 99.999%) with a backing pressure of 2 bar. The scattering surface used was HOPG, identical to the sample used in the O_2_-photodissociation calibration measurements. The laser beam was focused using a lens of 1 m focal length producing a 300 μm diameter beam waist at the center of the ion-optics. A detector gate width of 400 ns was used to ensure that NO^+^ ions with all *Y*-axis velocity projections were recorded, i.e., a “crush” image, within the optical slicing limits noted above.

The detector gate width is an important factor to consider if surface-VMI experiments are being run in the dc-slicing mode (i.e., gating the detector over a narrow time window of ion packet) as we have done for O_2_-photodissociation. Unlike the photodissociation experiments, the products of surface scattering were spread over a large spatial range as they traveled in all possible directions from the 10 mm-wide surface. Because of this NO^+^ ions were created far from the center of the ion-optics where they experienced slightly different electric fields from those at the center of the mapping region. This increased their flight time to the detector, decoupling ToF from the initial out-of-plane, *Y*-axis, velocity; N.B. ions are only created in the scattering plane due to optical slicing. This well-known phenomenon is one of the parameters that must be considered when VMI-ion-optics designs are optimized.^71^ Because of the relationship between detection point and scattering angle (detection further from the center was correlated with larger scattering angles), this correlated the ToF with the scattering angle. This effect is intrinsic to the VMI field curvature and not caused by the presence of the surface. It is only observable if ions are created far from the center of the ion-optics so would only be observed in a surface scattering experiment and not in a photodissociation experiment. Supporting Information Section SI-4 includes experimental and simulation results that show this effect. Thus, a broad detector gate width was required to detect NO ions created at all possible points along the laser propagation axis.

A common background signal is often observed in VMI experiments from molecules (from the molecular beam) that scatter from the ion-optics and other nearby surfaces and become thermalized before they escape this partially enclosed volume. Such molecules result in a 2-D Gaussian-like circular pattern centered around zero velocity in the images due to their Maxwell–Boltzmann distribution of speeds. These signals were exploited in the NS-VMI experiments to determine if the presence of the surface caused a shift in the location of zero velocity on the detector. By using a long image-acquisition time (30 min, 36,000 laser pulses) in experiments with the MB timing adjusted to fire directly after the previous laser pulse (i.e., ∼49 ms before the next laser pulse), complete thermalization was assured. The resultant images were fitted to determine the location of zero velocity for each L-S distance. The measured shifts (≤10 pixels, the same as those measured in the O_2_ photodissociation experiments) were consistent for each L-S distance. These measurements were repeated regularly. The same timings were also used to record a thermal-background excitation spectrum of NO by integrating the image intensity while varying the laser wavelength (see Figure 4b).

**Figure 4:**
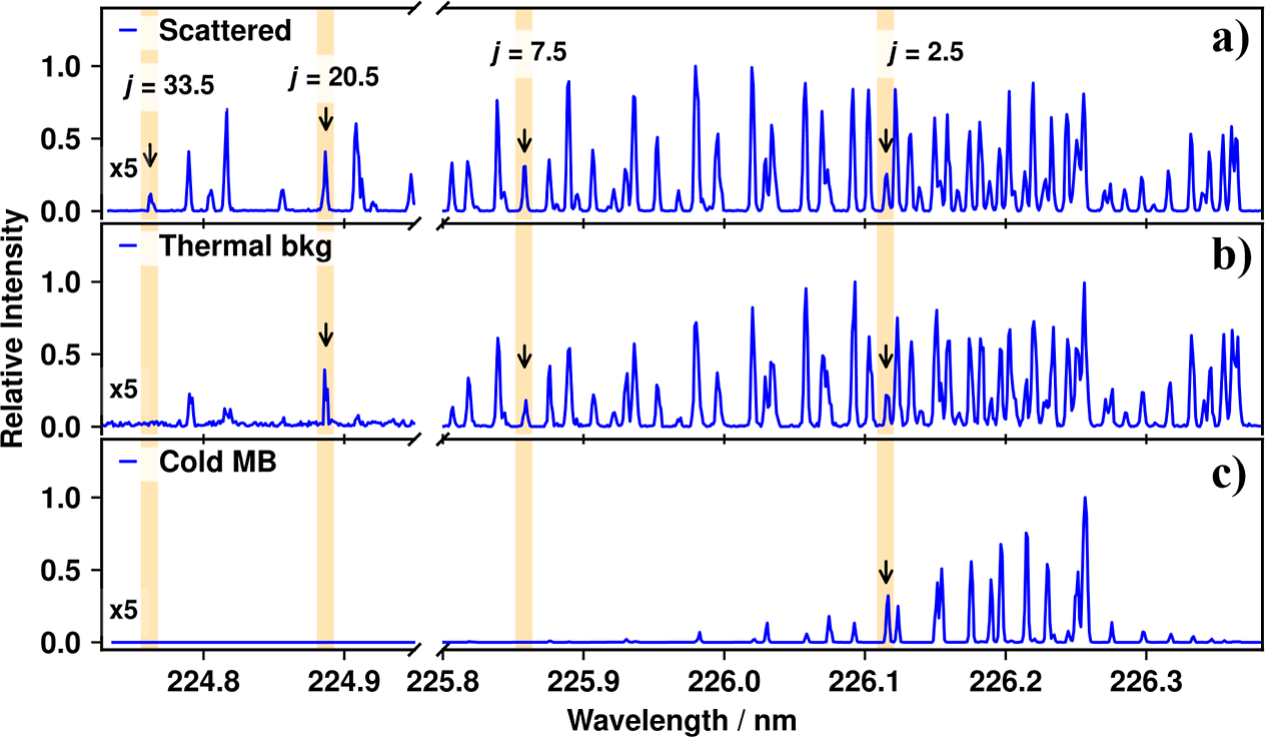
Individually normalized [1 + 1] REMPI excitation spectra of NO on the A^2^Σ^+^ – *X* ^2^Π (0,0) band: (a) surface-scattered NO; (b) the “thermal background”; NO molecules that have been thermalized through multiple collisions with the ion-optics and baffle; (c) NO in the incoming molecular beam in He carrier gas, with a typical modeled temperature of ∼15 K. The transitions that probe the rotational states that were chosen for detailed analysis, *j* = 2.5, 7.5, 20.5, and 33.5, are highlighted by light orange bars and black arrows. The low-wavelength ends of the spectra (224.72–224.94 nm) have had their intensities multiplied by five for ease of viewing the high-*j* signals.

In Figure 4, two wavelength regions of the NO REMPI excitation spectrum are presented for each of the three distinct types of signals visible in the scattering images. Panel (a) shows the molecules scattered from the surface; panel (b) shows thermalized molecules (recorded as described above), and panel (c) shows molecules in the incoming molecular beam. The spectra in panels (a) and (c) of Figure 4 were recorded using a laser pulse energy of ∼1 μJ, and the background spectrum on panel (b) used ∼8 μJ.

The in-going MB and surface-scattered (SS) spectra were obtained by summing the signal intensity in different regions of the image as a function of the laser wavelength, as shown in Figure 5a. Both MB and SS spectra were acquired simultaneously because the 10–90 width of the gas pulse (300 μs) is much longer than the laser pulse duration (∼5 ns). The laser is timed to fire coincident with the peak of the gas pulse (i.e., ∼80 μs from the 10th percentile of the gas pulse). In that time, molecules in the early part of the molecular beam have already scattered from the surface and returned to the ionization region. For a L-S distance of 5 mm, the incoming molecules take 2.8 μs to travel from the laser beam to the surface and scattered molecules going at 100 m s^–1^ would take an additional 50 μs to return to the probe volume.

**Figure 5:**
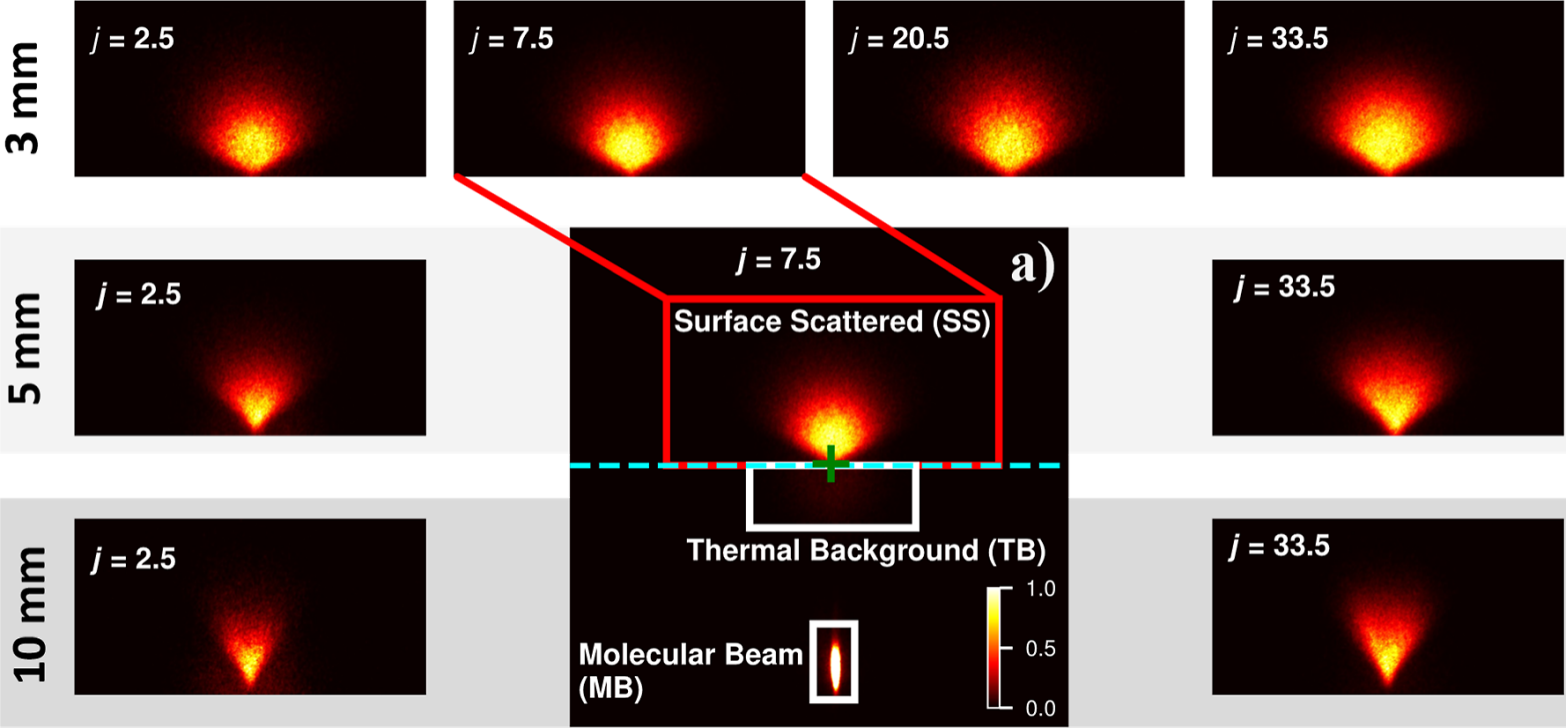
Raw images of NO scattering from HOPG. The main panel (a) is a full raw image showing three different regions of detected NO signal and other key features; the horizontal dashed cyan line bisecting the image separates the ions detected going toward the surface (lower half) and the ions detected traveling away from the surface, the green + on this line is the location of the zero-velocity point. The in-going NO MB is a bright elongated spot in the lower half of the image (velocity toward the surface). The red box in the upper part of the image contains the NO scattering from the surface as well as the upward-traveling part of the thermal background NO ions, the lower half of which are highlighted in a labeled white box (see the text for further details). Surrounding the main panel are raw images that have had the thermal background contribution subtracted and have then been cropped to show just the NO ions scattered from the surface for the four detected quantum states (*j* = 2.5, 7.5, 20.5, and 33.5) going across the figure, and for selected quantum states, the different L-S distances going down the figure. All images are independently peak normalized, with their peak intensities described by the color bar in panel (a); see the text for further details.

The SS molecules (traveling vertically upward in the lab frame) are defined as having positive-*Z*-velocity, i.e., above the cyan line in Figure 5a. The molecules in the MB have negative-*Z*-velocity and thus appear in the lower part of the image. The MB signal is clearly separated from the SS part of the image, thus, its spectrum was recorded using this part of the image. The SS signal is partially overlapped by the upper half of the weak thermalized-background signal. The lower half of this background is isolated (shown below the cyan dashed line in white rectangle in Figure 5a). To separate the SS and background signals, two spectra were extracted from the same sequence of images; one for the positive-*Z*-velocity ions in the red rectangular region (containing both the SS signal and the thermal background signal) and the other for negative-*Z*-velocity ions in the white rectangle in Figure 5a (which excludes the faster molecular-beam signal) that contains only the thermal background signal. Because of the circular symmetry of the background signal, subtracting the spectrum of the white region from that of the red region extracts the SS spectra.

The MB spectrum was comparable with a LIFBASE-simulated^72^ spectrum at a temperature of ∼10–15 K, with measurable populations only up to *j* ≤ 8.5. As we have used a focused laser beam with ∼1 μJ pulse energy, we expect some degree of saturation during excitation via the 1 + 1 ionization process, preventing a straightforward fit to extract a precise temperature; there is no guarantee, in any case, that the populations in MB source will be described correctly by a single temperature.

A large number of product rotational states were detected in the NO scattered from the HOPG surface, as shown in Figure 4a. This is consistent with recent calculations of the dynamics of NO scattering from HOPG,^32^ although we observe a greater range of rotational states experimentally. To span the potential scattering dynamics that might be present, we selected four states: *j* = 2.5, which represents only a small change in the rotational state from the initial *j* = 0.5 prepared in the molecular beam; *j* = 7.5 which is the most-populated state at room temperature (293 K); *j* = 20.5, which corresponds to a large change and has minimal population at room temperature; *j* = 33.5, which is one of the highest rotational states observed with substantial population in the scattered signal, and is negligible at room temperature. The relative populations of all rotational states at 293 K can be seen in Supporting Information Figure S13. Due to the congested nature of parts of the NO spectrum, the lines chosen [R_21_(2.5), R_21_(7.5), R_21_(20.5), and Q_1_(33.5) of the NO­(*A* ^2^Σ^+^ – *X* ^2^Π) (0,0) band] are spectrally isolated so that images can be recorded of a pure rotational state.

Comparing Figure 4a,c, out of the selected states only *j* = 2.5 is significantly present in the molecular beam, but all of the labeled states are readily detectable for the scattered molecules. The scattered distribution is clearly hotter than the thermalized sample, with different line intensities visible across the whole spectrum, and many higher rotational states being populated (including *j* = 33.5 which is not visible even in the thermal spectrum).

#### 3.2.2 Surface-Scattering Images

Background-subtracted scattering images are shown in Figure 5. The differing relative intensities of the four selected transitions in the scattered spectrum in Figure 4a implied that the acquisition of images of the same quality using the same laser fluence would take dramatically different durations for each state. Therefore, different laser fluences were used for each state to keep the data acquisition duration similar. This minimized the effects of any drifts in laser power or molecular-beam intensity over longer acquisition times. Typical laser powers used to record scattering images for each state were as follows: *j* = 2.5 (∼1 μJ), *j* = 7.5 (∼4 μJ), *j* = 20.5 (∼8 μJ), *j* = 33.5 (∼35 μJ). Typical images were acquired for ∼ 30,000 laser shots at a 20 Hz repetition rate.

To acquire a scattering image with background subtraction, the following procedure was used: (1) record a background image with the surface retracted from the ion-optics; (2) position the surface using the process described in the Experimental section 2.2; (3) record a surface-scattering image at the L-S distance defined in (2); (4) center the images recorded with and without the surface using separately recorded thermal-background images; and (5) subtract the background image from the surface-scattering image. Examples of images from steps 1, 3, and 5 can be seen in Supporting Information Figures S14–S16.

The background-subtraction procedure was used for all scattering images presented herein. A selection of the background-subtracted velocity-mapped images is shown in Figure 5, with a raw image of NO in the *j* = 7.5 state in Figure 5a. In this panel, as already described above, ions detected with a negative-*Z*-velocity component appear below the cyan dashed line, and those with a positive-*Z*-velocity component above it. The zero-velocity point is marked with a green cross. As also explained above, three features in the image are highlighted by labeled boxes. MB is the in-going NO molecular beam, which indicates the narrow angular collimation of the molecular beam. TB is the thermal background signal; it has its maximum intensity in this *j* = 7.5 image among the four quantum states recorded. The surface-scattered NO molecules are in the red box; it is this region that has been cropped out, with the thermal background contribution subtracted, for the other images in Figure 5. The images were taken at the peak of the MB gas pulse, as described in section 3.2.1. This provided sufficient time for incoming NO molecules earlier in the molecular-beam pulse to reach the surface and for those scattered molecules with speeds above ∼100 ms^–1^ to return to the ionization region.

The images for different *j* states across the top row of Figure 5, taken at an L-S distance of 3 mm, show little difference in their raw state. In contrast, the first and last columns (*j* = 2.5 and 33.5, respectively) show how the observed scattering changes significantly with L-S distance. The effects of tunnel vision can be seen directly in these images; the larger L-S distances have apparently narrower angular-scattering distributions. This is also the case for *j* = 7.5 and *j* = 20.5, not shown here; Supporting Information Figure S17 shows a full comparison of all the quantum states and L-S distances recorded.

We wish to present the measured distributions as scattered flux as a function of speed and angle in polar coordinates. First the raw Cartesian image of measured pixel intensities *I*(*z, x*), which is proportional to the number density of NO^+^ ions (ρ_NO_^+^) at points on the detector, is transformed to polar coordinates, *I*(*r,* θ_f_)*,* according to

$$\iint_{R_{xz}}I\left(x,z\right)dxdz=\iint_{R_{r\theta_{f}}}I\left(r,\theta_{f}\right)rdrd\theta_{f}$$ 1

using the relation *z = r* sin θ_f_ and *x = r* cos θ_f_, where *r* is the radial pixel coordinate, measured from the origin at the zero-velocity pixel.

To improve the signal-to-noise ratio, the intensity is summed over wedges with angular resolution of Δθ_f_ = 3° and radial resolution Δ*r* = 1 pixel. The averaged intensity within each wedge is then calculated as

2 $$I\left(\Delta r,\Delta \theta_{f}\right)=\frac{\sum_{r}^{r+\Delta r}\;\sum_{\theta_{f}}^{\theta_{f}+\Delta \theta_{f}}I\left(r,\theta_{f}\right)r\Delta r\Delta \theta_{f}}{n_{pix}}$$

where *n*_pix_ is the number of pixels within a wedge. Dividing by *n*_pix_ accounts for the increase in the area of the wedge as *r* increases. The factor of *r* here is the Jacobian from eq 1. This procedure is applied over the entire scattering region, *r* = 0,1,2, ..., 500, and θ_f_ = −90, −87, −84, ..., 90°.

Subsequently, a density-flux correction was applied, to account for the very well-known fact that faster molecules spend less time in the laser interaction region than slower ones, which results in the detected ion density being inversely proportional to molecular speed and hence to *r*. The flux of scattered products in polar coordinates (*v*, θ_f_) is

3 $$I_{flux-corr}\left(\Delta v,\Delta \theta_{f}\right)=\nu_{cal}rI\left(\Delta r,\Delta \theta_{f}\right)$$

where ν_cal_ is the speed-to-pixel calibration factor and the factor of *r* here achieves the density-flux correction. Using this correction, we have plotted the density-flux-corrected intensity of scattered products from the HOPG surface for *j* = 33.5 as a function of speed and θ_f_ in Figure 6. Equivalent plots for the other recorded states can be found in Supporting Information Figure S18.

**Figure 6:**
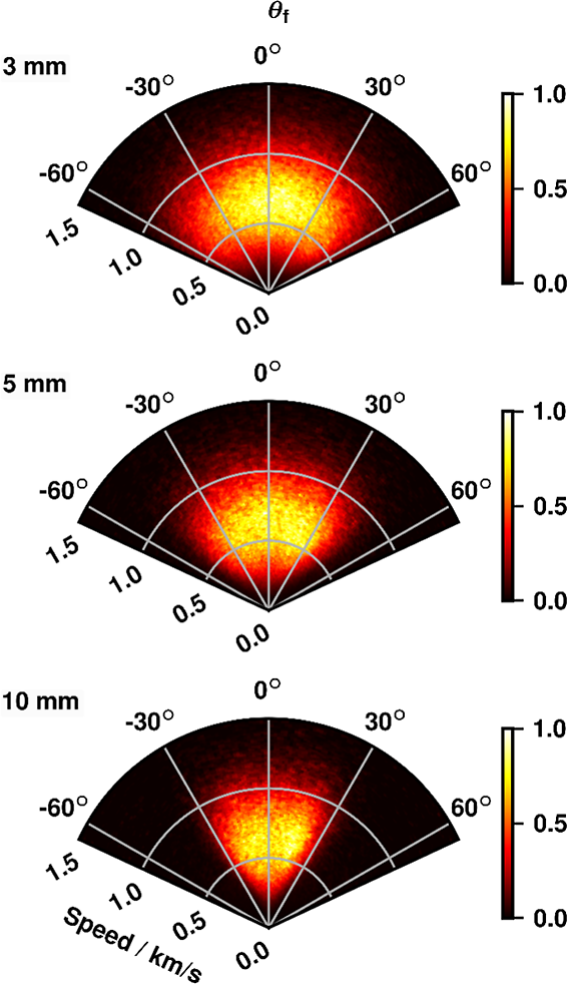
Density-flux-corrected images of NO (*j* = 33.5) scattered from an HOPG surface at three L-S distances. Bottom panel: L-S = 10 mm, the observed scattering has a low angular range fwhm ≈ ±30° about the surface normal. Middle panel: L-S = 5 mm, the observed scattering has broadened to fwhm ≈ ±45°. Top panel: L-S = 3 mm, the broadest observed scattering, with fwhm ≈ ±55°.

For all the L-S distances, the flux distributions exhibit a peak between 500 ms^–1^ and 1000 ms^–1^, with negligible to no intensity observed above 1500 ms^–1^; hence the plots in Figure 6 are truncated at this speed. This indicates significant energy exchange upon collision with the surface as the MB average speed for these measurements was 1780 ms^–1^. This is consistent over all the L-S distance, including at 3 mm, despite the reduced speed resolution determined in the calibration experiments. This indicates that essential speed-distribution information remains preserved in the acquired data.

As the L-S distance decreases, the full width half-maximum (fwhm) of the observable scattering angle increases markedly, from approximately ±30° at 10 mm to ±55° at 3 mm. This reflects the corresponding changes already noted in the nondensity-flux-corrected images in Figure 5. A more quantitative analysis of the speed and angular distributions extracted from these plots is presented in the following section.

#### 3.2.3 Illustrating the Impact of L-S Distance on Measured Speed and Angular Distributions

Here, we present speed and angular distributions to demonstrate that the NS-VMI approach is capable of providing physically plausible data that evolves as expected as a function of L-S distance; a more detailed analysis connecting the observed scattering to underlying mechanisms will be provided in future work.

To examine the speed distribution as a function of L-S distances, the speeds were integrated over all the angles in the scattering region. These speed-dependent flux distributions are illustrated in Figure 7 for four quantum states (*j =* 2.5, 7.5, 20.5, and 33.5) at the L-S distance of 5 mm; results for all L-S distances can be seen in Supporting Information Figure S19. The distributions were fitted with a phenomenological two-component function, as has been used in this field by others (e.g., by Neumark and co-workers)^19,20^

4 $$f_{1}\left(\nu \right)\propto \nu^{3}\exp\left(-\frac{m\nu^{2}}{2RT_{s}}\right)+f_{2}\left(\nu \right)\propto \nu^{3}\exp\left(-\frac{m{\left(\nu -\nu_{2}\right)}^{2}}{2RT_{2}}\right)$$

where ν is the speed, *m* is the mass of the scattered species, and *R* is the universal gas constant. *T*_s_ is the temperature of the surface (293 K for the present experiments), *T*_2_ is the width parameter of the second component, and ν_2_ is the speed offset of the second component. Suitable factors were included to ensure the two components were independently normalized. The fit returned the weighted contributions of each. This function is used as it provides a good fit to the data, and the two components allow a straightforward way to compare the speed distributions of the different quantum states detected.

**Figure 7:**
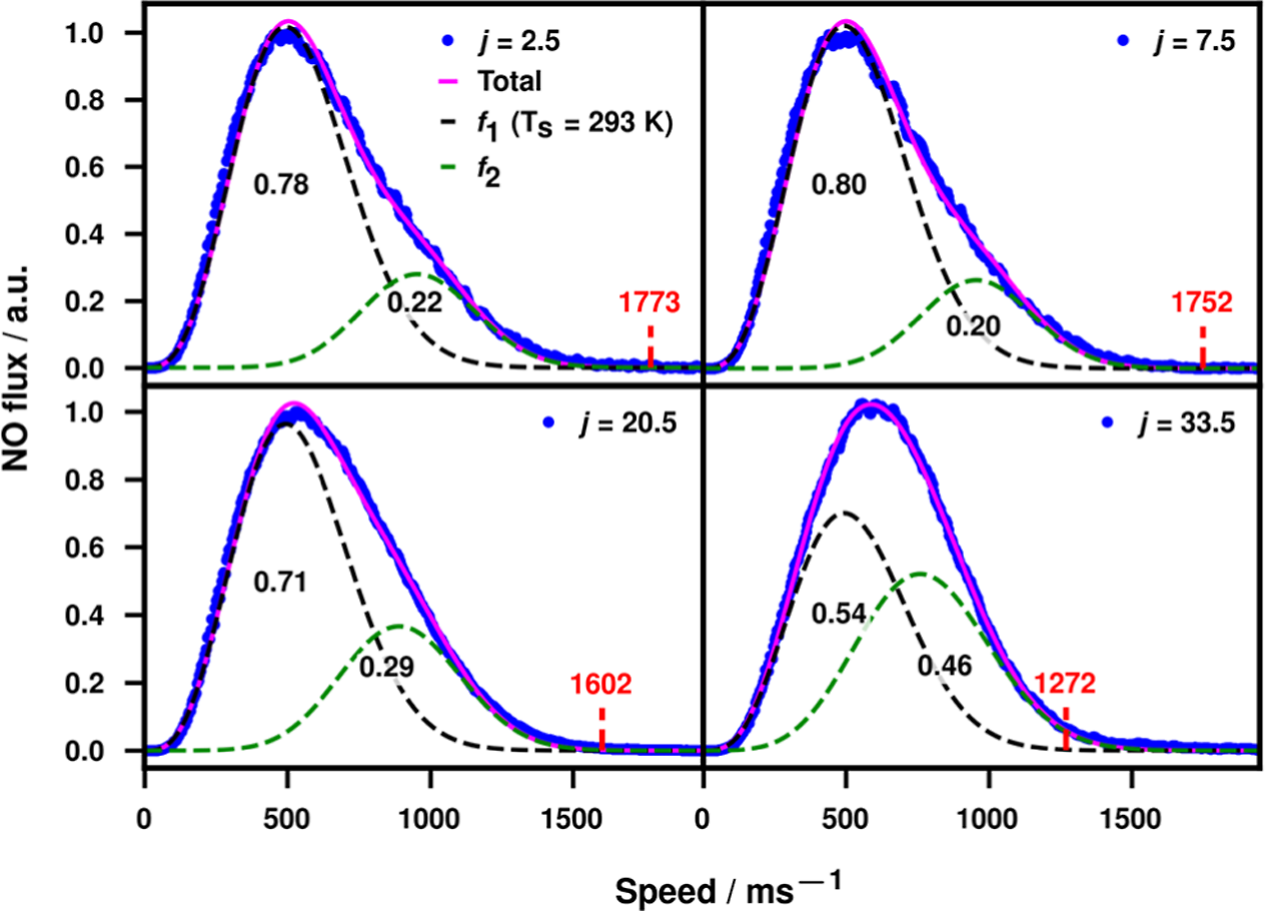
Angularly integrated distributions of flux as a function of speed for all recorded quantum states at L-S = 5 mm. Each panel shows the experimental data as blue circles with a two-component fit to the data, defined by eq 4 in the text, shown as a magenta line. The *f*_1_ component of the fit is shown as a black dashed line; and the *f*_2_ component is the green dashed line, the relative areas of the two components are shown in text under each curve (see the text for details). A speed cutoff, as calculated using the mean MB speed and energetics of the scattering into the probed rotational state, is shown as a labeled dashed vertical red line (see the text for more details).

The quantum-state-dependent measurements allowed us to examine whether the observed changes in speed distribution with quantum state could be explained solely based on energy conservation. The maximum possible velocity of scattered products was calculated for each of the four probed states. In this calculation, it was assumed that the initial rotational state of the colliding NO molecules is *j* = 0.5 (a good assumption as this is 81% of the incoming molecular beam) and that the speed of the incoming NO is the average of the MB speed (1780 ms^–1^, directly measured from the velocity-map images). The maximum speed is then calculated simply from the energy balance of the initial kinetic energy minus the rotational excitation energy. The resultant maximum speeds for the observed rotational states *j* are listed in Table 2 and are shown as labeled vertical dashed red lines in Figure 7.

**Table 2:** Measured and Calculated Values for Flux as a Function of Speed Derived from Figure 7, along with Relative State Populations Calculated for a Thermal NO Sample

*j*	2.5	7.5	20.5	33.5
calculated maximum speed/ms^–1^	1773 ± 115	1752 ± 117	1602 ± 128	1272 ± 161
peak speed/m s^–1^	502	502	522	589
proportion of fitted *f*_1_ component	78%	80%	71%	54%
relative population for a bulk sample at 293 K (see Supporting Information Figure S13)	0.59	1.0	0.12	5.5 × 10^–4^

The peak speeds seen in Figure 7 (listed in Table 2) show the large exchange of translational energy during the collisions with the surface. As *j* increases, the peak speed increases, showing a slight propensity for conservation of a higher proportion of the initial translational energy for higher rotational states. However, it can also be seen that the high-speed edge of the distribution drops more steeply with increasing *j*, consistent with more-impulsive scattering. The proportion of the distribution that is fitted by the first (surface-temperature-related) component *f*_1_ of the fit decreases either side of the *j* = 7.5 result, nominally the most-populated state in a surface-temperature Maxwell–Boltzmann distribution. The proportions of *f*_1_ for the observed rotational states *j* are listed in Table 2 alongside the relative populations for a thermalized 293 K bulk sample of NO. It is worth stating, in passing, that these relative populations highlight the danger of overinterpretation of fitting functions that contain a Maxwell–Boltzmann component; the fraction of thermal population in *j* = 33.5 is only 0.05%, but the fraction of *f*_1_ fitted by eq 4 was over 50%. Slow moving *j* = 33.5 molecules were either formed directly in a single collision, or they were the result of secondary collisions that removed translational energy from a highly rotationally excited molecule without significantly changing its rotational state.

Inspection of the angle-integrated speed distributions for each quantum state at each of the L-S distances, shown for *j* = 33.5 in Supporting Information Figure S20, reveals little or no variation as a function of L-S distance. In other words, bringing the surface near to the laser beam does not introduce any distortions in the measured speed distributions, integrated over all detected angles. The trend of a reduced *f*_1_ component on either side of *j* = 7.5 at L-S distance of 5 mm was also consistent at the other two distances. These results indicate that the speed resolution loss at 3 mm, identified in our calibration experiment, or other potential distortions do not compromise the faithful extraction of scattered-speed information.

To analyze the angular distribution of the scattered products and establish if they are meaningfully correlated with speed, NO fluxes over three speed ranges (0–500, 500–1000, 1000–1500 ms^–1^) were summed and plotted separately as a function of scattering angle, i.e.

5 $$I\left(\theta \right)=\sum_{\nu_{initial}}^{\nu_{final}}I_{flux-corr}\left(\nu ,\theta \right)$$

These distributions are shown for the *j* = 7.5 scattering images in Figure 8.

**Figure 8:**
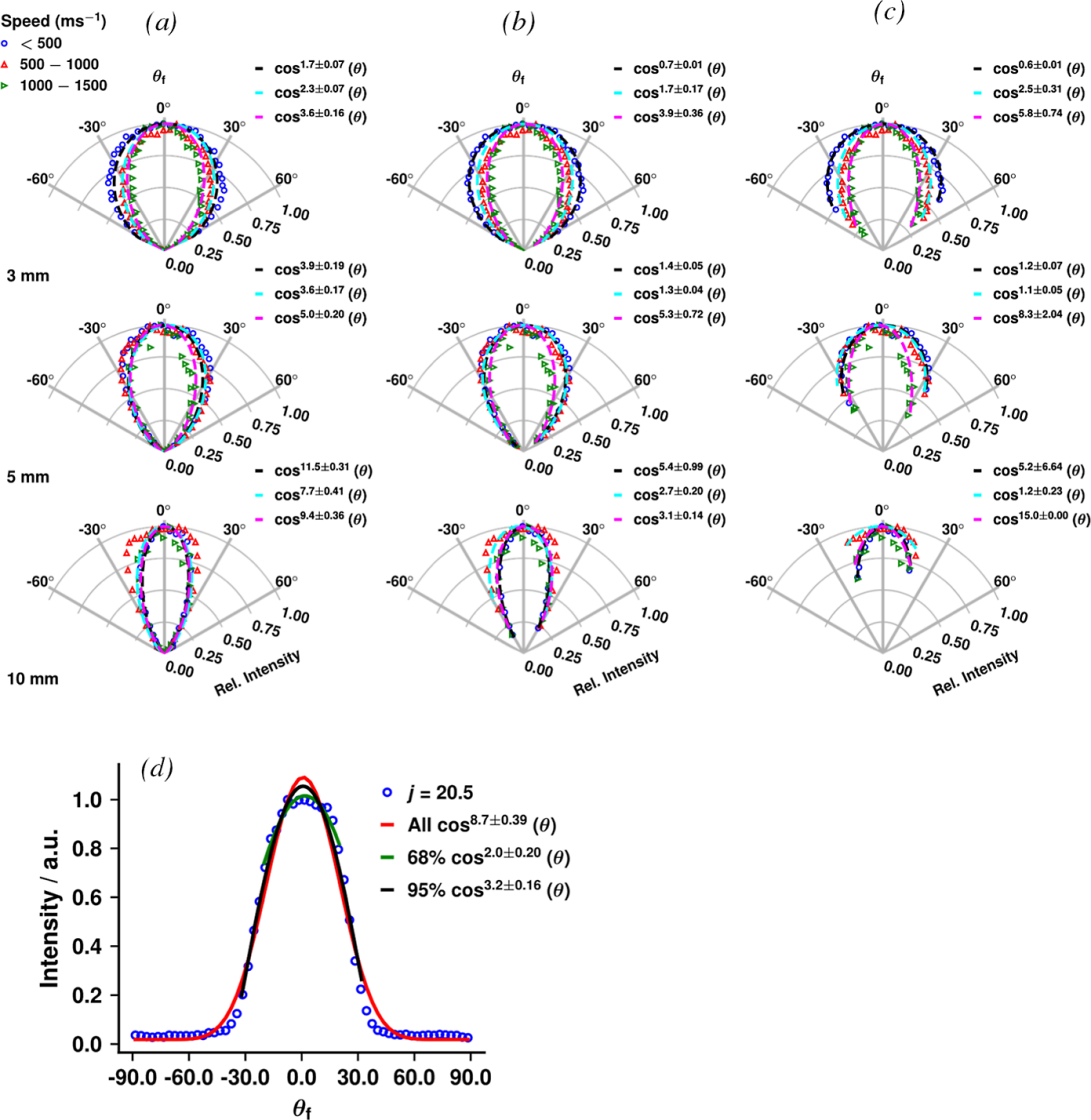
Experimental angular distributions for *j* = 7.5 with fits, as defined in eq 6 in the text, at each recorded L-S distance (top row: L-S = 3 mm, second row: L-S = 5 mm, third row L-S = 10 mm). The plots show the angular distributions for each of three speed regions: speeds below 500 ms^–1^ (blue circles); speeds between 500 and 1000 ms^–1^ (red upward triangles); and speeds between 1000 and 1500 ms^–1^ (green sideways triangles). The experimental data points in each column are identical; however, the angular range of data plotted and used for the fits changes: Column (a) displays the full range of experimental data. Column (b) displays the central 95% of the geometrically possible scattering intensity for each L-S distance: top ±64°, second ±51°, third row ±32°. Column (c) displays the central 68% of the geometrically possible scattering intensity for each L-S distance: top row ±50°, second ±35°, third ±20°. The fitted *n* values are shown with their uncertainties for each of the speed ranges next to each plot. Panel (d) shows the experimental angular distribution over all speeds for *j* = 20.5 at L-S = 10 mm (blue open circles) in rectilinear coordinates with fits to eq 6 for: The full angular range of the data (red line); the data range that represents the central 95% of the geometrically possible scattering intensity ±32° (black line); and the data range that represents the central 68% of the geometrically possible scattering intensity ±20° (green line). See the text for further details.

The cosine power function is commonly used to parametrize the angular distribution of surface-scattered products.^5,11,57^ A small exponent indicates a broad, more-isotropic distribution, whereas a larger exponent corresponds to a more-sharply peaked, anisotropic distribution. For our analysis, the angular intensity distribution *I*(θ_f_) for each speed range was fitted using a cosine power function as follows

6 $$I\left(\theta_{f}\right)\propto \cos^{n}\left(\theta_{f}\right)$$

A value of *n* = 1 corresponds to a spherical distribution such as that from a Knudsen source. The first column (a) in Figure 8 shows the full range of the experimental angular data for the *j* = 7.5 state. Cosine fits are included for each of the three different speed ranges, with separate plots for each of the L-S distances measured. In the fits to eq 6, *n* was limited to the maximum value of 15; this limit was only reached for one data set and increasing the limit did not lead to an improved fit or reduced errors.

The data in Figure 8 once again clearly show the influence of tunnel vision, with the L-S = 10 mm results (third row) showing the narrowest angular distributions for all speed ranges, followed by the L-S = 5 mm (second row), with the L-S = 3 mm (top row) showing the broadest distributions. The reduction in the exponent *n* values of the fits reflects the increase in the acceptance angle as the L-S distance is reduced. Angular fits to the full set of data for all the other *j* states measured are qualitatively similar: they are presented in Supporting Information Figure S21. Note that the angular ranges shown in Figure 8 compare favorably with our modeled predictions that are shown in Figure 1. For L-S = 10 mm, an angular range of ±32° about the surface normal was predicted compared with ±35° for the experimental data; for L-S = 5 mm, the prediction of ±51° is very close to the ±50° measured; and for L-S = 3 mm, the modeled ±64° is comparable with ±60° recorded.

The surface scattering process is the same for all experiments run at different L-S distances, so the changes in recorded angular range must be due to the tunnel vision effect. This will affect the fitting of the angular distributions, as for many of the (especially longer, 5 and 10 mm) L-S distances, the fits will be strongly influenced by the many data points that have zero value. This is illustrated in Figure 8d where rectilinear coordinates are used to plot the experimental angular distribution over all speeds for *j* = 20.5 at L-S = 10 mm to highlight the large majority of the data points that have effectively zero intensity. To ensure that our fits were not influenced by the large number of near-zero baseline points, additional fits were made; the angular ranges for these fits were restricted to regions that we calculated using the geometry of the experiment (see Figure 1 and Supporting Information-1). The reduced angular ranges encompassed the central 95% and 68% of the surface-dosing-weighted geometrically possible scattering intensity. The “95%” angular region eliminates the effect of the baseline on the fitting, shown by the black line in Figure 8d. The “68%” region removes any possible effects caused by ions that have been recorded in the images but were created at, and beyond, the limits of the velocity-mapping region; such ions were detected but not mapped with high enough resolution. This can only happen for the highest scattering angles at each L-S distance. The results of these reduced angular range fits are presented in Figure 8b,c.

The *n* values in Figure 8d change dramatically when the angular fitting range is reduced, showing that a long, near-zero baseline can strongly bias results, and/or that the cosine power function may not be suitable for such fitting. Examining the changes in *n* across the rows in Figure 8a–c shows that such dramatic changes were not isolated to the single example in panel (d). The other important conclusion that can be drawn is that even restricting the analysis range of data taken at too great an L-S distance will not allow the extraction of the same fitted parameters as those taken when the surface is near the ionization source. The overall trends in the fitting can be better examined by compiling the fitted *n* values for all measured quantum states and plotting them as a function of speed range, as shown in Figure 9.

**Figure 9:**
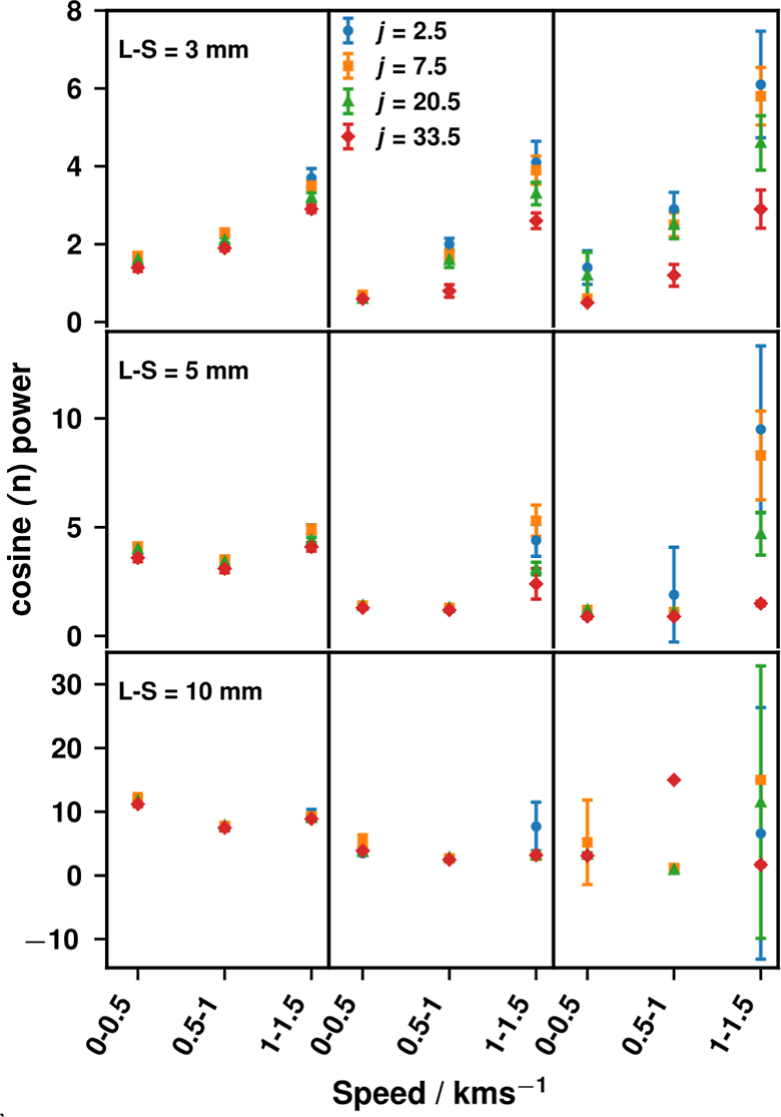
Exponent *n* obtained from the angular distribution fit using eq 6 is plotted as a function of the speed region for each rotational state at L-S distances of 10, 5, and 3 mm (in rows from bottom to top). The fitted *n* values for each rotational state are shown (*j* = 2.5, blue circles; *j* = 7.5, orange squares; *j* = 20.5, green triangles, and *j* = 33.5, red diamonds) with the fit uncertainties shown as error bars. Column 1 displays the results of fitting to the full range of experimental data. Column 2 displays the results of fitting to a data range that represents the central 95% of the geometrically possible weighted scattering intensity: bottom row ±32°, middle ±51°, top ±64°. Column 3 displays the results of fitting to a data range that represents the central 68% of the geometrically possible weighted scattering intensity bottom row ±20°, middle ±35°, top ±50°.

The plots in Figure 9 show a summary of the angular distribution fits for all speed ranges at all three L-S distances for all four states measured, with the columns showing the results of fitting to different angular ranges. The most visible consequences of fitting to reduced angular ranges are the change in magnitude of the fitted *n* value, and a large increase in the uncertainty of the fits, especially for the highest speed range. The smaller uncertainties shown in column 1 of Figure 9 are due to the fit being dominated by the zero-intensity points which the function can reproduce very well. For the reduced range fits in columns 2 and 3 of Figure 9, the large error bars are due to both the cosine power function being unsuitable for fitting the smaller number of data points and the lack of baseline points driving the fit quality. By excluding the baseline points for the “95%” data, almost all values of *n* decrease, demonstrating the influence that such zero-intensity points have on the fitting process.

The most significant point highlighted by Figure 9 is that at L-S = 3 mm, for all the states measured the exponent *n* clearly indicates systematically narrower angular distributions for higher speeds. This is true across all the different data ranges used for fitting; only at L-S = 3 mm, is there evidence of a correlation between the speed and scattering angle distribution. There are good physical reasons to believe that this clear correlation is real, reflecting the more-impulsive scattering expected into higher rotational states. However, the correlation is lost at L-S = 5 and 10 mm. In principle, the combination of the different angular detection ranges as a function of L-S distance and the variation of the scattering-angle distribution with speed would imply that we should observe different *angle-integrated* speed distributions as a function of L-S distance. Clearly, as discussed above and shown in Figures S19 and S20, we did not. We believe that this is the chance consequence of canceling contributions as the L-S distance is varied in this more highly averaged, angle-integrated, measurement, which can only be disentangled at the smallest L-S distance with its wide range of detectable angles. We conclude that not only does tunnel vision clearly lead to artificial narrowing of angular distributions overall, due to incomplete sampling at the larger L-S distance measurements, but that it may also lead to more subtle losses in dynamically significant information.

## 4 Conclusions

A new instrument has been constructed that allows the near-surface ionization and velocity-mapping of products from gas-surface-scattering experiments. Optical slicing allows NS-VMI to directly image the scattering plane. Effective velocity-mapping is shown to still be possible even with the electric-field-perturbing and symmetry-breaking effects of a dielectric surface being placed inside the velocity-mapping region. The capabilities of the NS-VMI spectrometer have been demonstrated at a series of distances ≤10 mm (all significantly smaller than previous work)^57,58^ between the laser and the surface with measurements of NO scattering from an HOPG surface into multiple quantum states. The quantum-state-specific images have been analyzed in terms of both their speed and angular distributions to demonstrate how well the dynamical information that they contain is recovered as a function of the laser-surface distance. By introducing the ability to vary this distance, these experiments have demonstrated that “tunnel vision” is a real phenomenon in gas-surface-scattering experiments which use laser probes. They have also shown that the path to beating tunnel vision in such experiments is to directly image the 2D scattering plane using an ionization source as near to the surface as possible.

## Supplementary Material



## Notes

Detection geometry; additional apparatus details; SIMION simulations of VMI and SMI; and results for all product quantum states at all laser surface distances (PDF)
