# Supplementary material for: Beating Tunnel Vision: Near-Surface Velocity-Map Imaging
Source: J Phys Chem A. 2026 Feb 17;130(8):1713–27. doi: 10.1021/acs.jpca.5c08379 (PMC12951571; doi:10.1021/acs.jpca.5c08379)
Supplement: Supplementary file 1 [file jp5c08379_si_001.pdf]

Beating Tunnel Vision: Near-Surface Velocity-Map Imaging – Supporting Information

Nitish Pal,[1] Preeti M. Mishra,[2] Paul D. Lane,[1] Matthew L. Costen,[1] Kenneth G. McKendrick[1] and Stuart J. Greaves[1].*

AUTHOR ADDRESS

[1]Institute of Chemical Sciences, Heriot-Watt University, Edinburgh EH14 4AS, United Kingdom

[2]Department of Physics, Government Women's College Keonjhar, Mandua, 758001-Odisha, India

*Author to whom correspondence should be addressed: s.j.greaves@hw.ac.uk

TABLE OF CONTENTS

SI-1. Geometry of Detection S2

SI-2. Experimental Details S4

SI-2.1 Apparatus S4

SI-2.2 HOPG surface S6

SI-2.3 Molecular Beam Timing S7

SI-3. SIMION Simulations S8

SI-3.1 Velocity Map Imaging S8

SI-3.2 Spatial Map Imaging S10

SI-3.3 Calibration of VMI S12

SI-3.4 Calibration of VMI from NO_2 for NO species in scattering geometry: S14

SI-4. ToF of Ions Outside the Mapping Volume S16

SI-5. Relative Populations of NO S18

SI-6. Scattering Results S19

SI-6.1. Background Subtraction S19

SI-6.2. Background Subtracted Scattering Images S22

SI-6.3. Speed and Angular Distributions S23

SI-1. Geometry of Detection

The geometry of the surface and ionization laser in the experiment imposes restrictions on the surface-scattering angles that can be observed in any given surface velocity-map imaging (VMI) experiment. As described in the main text there are several factors that can affect the observable surface scattering angles; for a given combination of surface size, distance from laser to surface, and size of the ionization/velocity mapping region only product molecules with particular trajectories can leave the surface and pass through the ionization/mapping region. The range of detectable angles was determined by a straightforward numerical simulation that randomly chose points on the surface and in the mapping volume and calculated the projection of the scattering angle in the scattering plane. By repeating the process it is possible to create a histogram of the geometrically accessible scattering angles for given surface and laser/mapping region dimensions and the distance between the two. The surfaces used in our experiment were 10×1 mm, the laser-surface (L-S) distance was controllable and the distances used were 10, 5, and 3 mm. The laser beam had a beam waist of 300 μm, and the effective mapping length was determined to be 10 mm by SIMION simulation, see SI-3.1 below.

Using the geometric parameters above a 1° binned histogram of the possible scattering angles for each L-S distance was created by randomly sampling 1 million combinations of surface and laser positions. At the extreme edges of these symmetrical histograms there is a very low count rate as some scattering angles can only be measured if a molecule is scattered from one extreme edge of the surface and detected at the opposite extreme edge of the mapping volume. This effect is compounded by the effect of surface dosing from our molecular beam (MB); such dosing is not uniform across the surface but can be measured experimentally by spatially mapping the incoming molecular beam, see SI-3.2 below. The dosing of the surface is gaussian in nature where incoming molecules preferentially hit the center of the surface. As the most extreme scattering angles detectable rely on molecules striking the edges of the surface the low dosing of these parts of the surface means that they are even less likely. This was simulated by weighting the scattering angle histograms by a normal distribution (defined by the molecular beam parameters) dependent on the surface location of each random geometry. An example of a calculated geometric histogram is shown in Figure S1 below.

The angular ranges presented in Figure 1 of the main text represent the central 95% of the possible scattering intensity in the surface dosing weighted histograms. A summary of these values is presented in Table S1 along with angular ranges of the central 68% for the same L-S distances. Also presented in Table S1 are the 'out-of-plane' angular ranges that show the effect of optical slicing in limiting the scattered product ionization to the scattering plane, see section B of the Results and Discussion section of the main text.

Table S1 Scattering plane and out of plane angular ranges of the central 95% and 68% of the cumulative intensity of the surface dosing weighted geometric histograms, see text for details.

L-S distance / mm	95% in plane	95% out of plane	68% in plane	68% out of plane
3	±64°	±9.7°	±50°	±6.4°
5	±51°	±5.85°	±35°	±3.8°
10	±31.5°	±3°	±19.7°	±1.9°

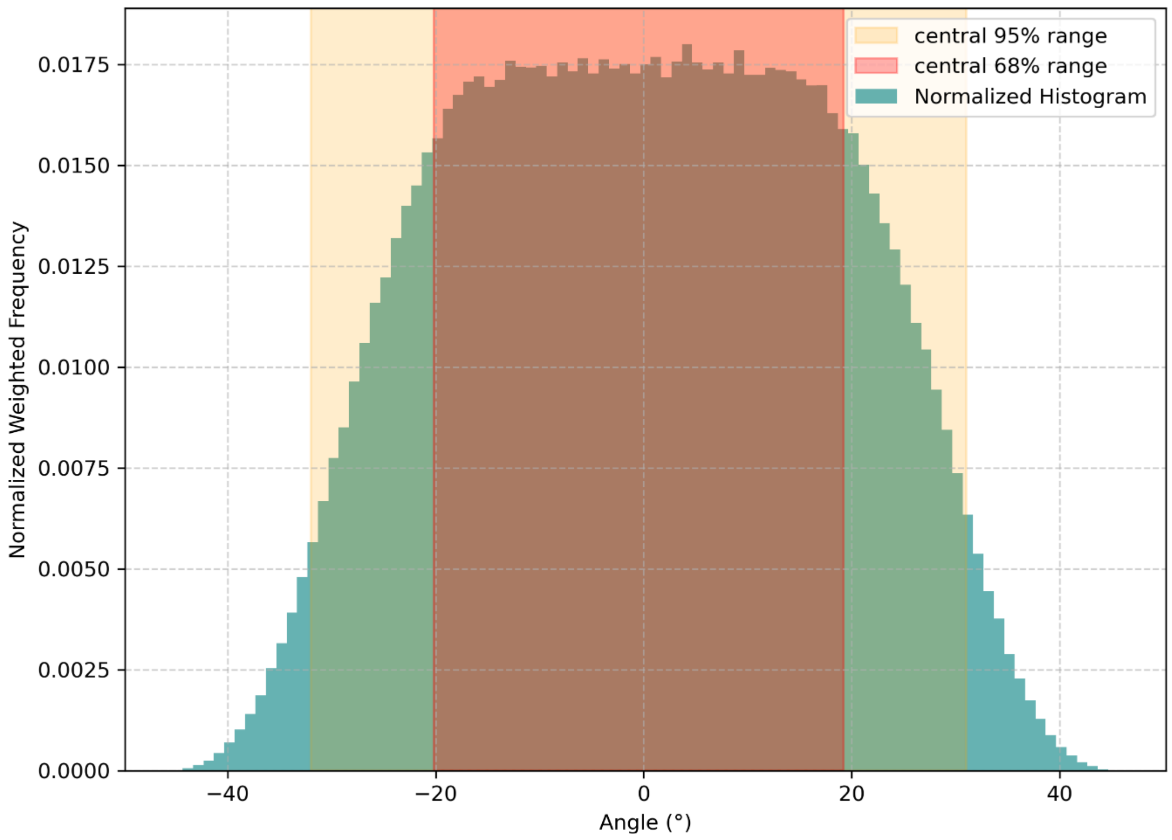

Figure S1. *Histogram of geometrically accessible scattering angles for a laser surface distance of 10 mm that has been weighted by a surface dosing gaussian with a full-width half-max of 4 mm, see text for details. The central 95% and 68% cumulative intensity is indicated with yellow and orange shaded regions respectively*

SI-2. Experimental Details

SI-2.1 Apparatus

The experimental apparatus (depicted in Figure S2a) consists of five differentially pumped regions: molecular beam source, ionization, time-of-flight (ToF), detector, and the load-lock chamber, with base pressures of 1.6×10^{-8}, 1.6×10^{-8}, 3.4×10^{-8}, 1.17×10^{-8}, and 1.14×10^{-8} mbar, respectively. All regions are pumped by turbomolecular pumps: molecular beam source & ionization region by a Pfeiffer ATP 2300M; ToF & detector regions by two Edwards nEXT85D pumps; and the load-lock by a Pfeiffer HiPace 700. All turbomolecular pumps are backed by dry scroll pumps (3 × Edwards nXDS15iC).

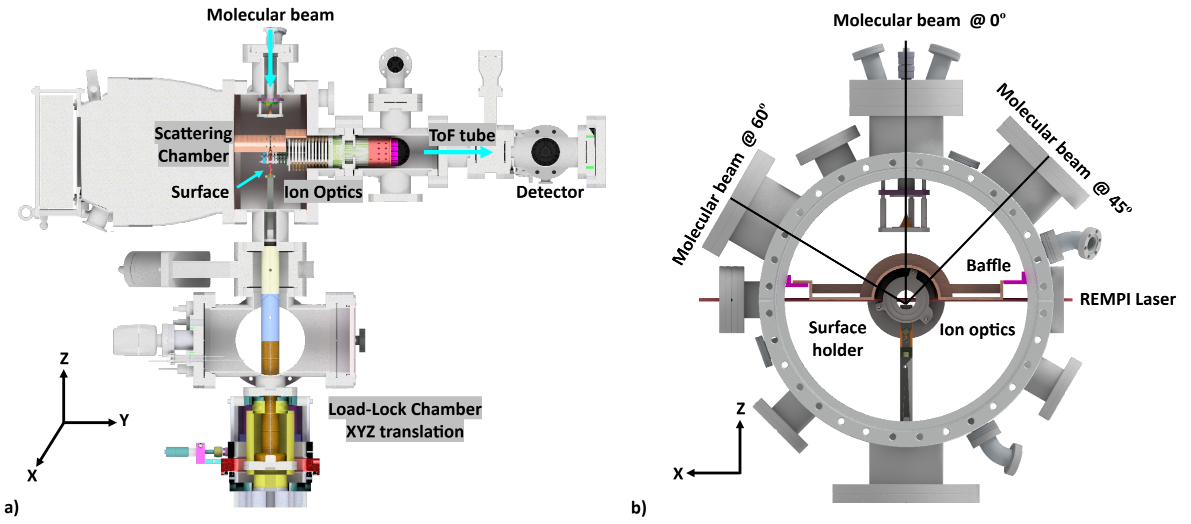

Figure S2. A schematic of NS-VMI apparatus. a) Half section view along the ToF of the experimental setup depicting the scattering chamber separated in two: the molecular beam source region and the interaction region. The distance between the surface normal skimmer and the ionization region is 73 mm along the Z axis. The ToF distance from the interaction region to the detector is 686 mm. b) Scattering chamber in the XZ (scattering) plane with general valve assembly mounted on the surface normal port. Also shown are the MB ports at 45 and 60 that enable scattering experiments at these incident angles. See text for details.

The main chamber (based on a DN250CF cylindrical tube with a horizontal length of 150 mm) sits at the heart of the experiment and is connected to other regions of the experiment: Vertically below the main chamber is the load-lock chamber which can be isolated by a DN100CF gate-valve; the surface is introduced and positioned via the load-lock chamber using an XYZ translator (LewVac M-XYZ-12-406-63CF). Horizontally along the Y-axis from the main chamber is the time-of-flight (ToF) chamber that mounts the ion optics (and attendant HV electrical feedthroughs) and connects to the detector chamber. The ToF is connected to the main chamber via a DN100CF flange, the size is reduced to a DN63CF tubulation connected to a DN63CF gate-valve connected to the DN63CF detector chamber. The DN63CF gate valve is used to isolate and maintain vacuum in the detector chamber which houses the 40 mm diameter in-vacuum position sensitive detector (Photek VID240, GM-MCP-2, P46 phosphor).

The ToF tube and detector chamber are mounted on rails, which allows the ion optics to be slid out of the scattering chamber for maintenance or electrode replacement.

The main chamber itself is split into two regions with a horizontal baffle, as shown in Figure S2b. The baffle is formed of a horizontal metal plate with a semicylindrical center section that encloses the ion-optics. Above the baffle there are three DN75CF ports for mounting pulsed valve assemblies at different angles relative to the surface normal (0°, 45°, and 60°), in the current work only results generated when using the normal incidence (0°) are presented. This upper baffled region of the chamber forms the molecular beam source region. There are two additional viewports either side of the surface-normal port that provide visual guidance for placing the surface within the ion optics during insertion or retraction. More precise alignment was carried out with a diode laser and camera to check the laser–surface (L–S) distance.

Each pulsed valve assembly in the source region consists of a general valve (Parker Series 9) and a skimmer (Beam Dynamics) with a 0.5 mm diameter orifice, separated by 20 mm. These components are mounted on a DN75CF flange using a cage structure made of three stainless steel rods that allows in-vacuo adjustment of the nozzle skimmer distance. Before installation, the valve and skimmer are aligned with a diode laser to ensure accurate MB propagation.

Below the main chamber baffle (the ionization region) are two viewports for laser entrance and exit on the horizontal axis, as shown in Figure S2b) a red line labelled REMPI laser shows the laser path. Also below the baffle are two DN40CF ports opposite the 45° and 60° ports to allow in-situ nozzle and skimmer alignment. These ports are in addition to the connection to the load-lock chamber that sits vertically below the normal incidence port. Two pressure gauges (Edwards AIM200), one above and one below the baffle (mounted via DN16CF elbows), are installed to measure the pressure in the source and ionization regions.

The velocity-map imaging (VMI) ion-optics are mounted from a double sided DN100CF flange that forms part of the ToF tube. A sectioned view of the ion optics can be seen in Figure S3; this is an expanded view of that seen in Fig. 2 of the main paper. The ion-optics are mounted to the DN100CF via the final grounded electrode in the stack shown in green. From the lens electrode to the grounded electrode the ion-optics have a larger outer diameter, the voltages on these electrodes are controlled via a resistor chain that smoothly decreases the voltage from the lens to ground in equal steps. The ion-optics closer to the interaction region are smaller in diameter (see main paper for discussion of this design), and the repeller electrode is translucent in Figure S3 to show the laser path. The voltages on the ion-optics were controlled using a high voltage power supply (iseg EDS F1 30p (16 x 3kV channels) power supply mounted in a Mpod Micro 2 controller). The figure also shows the deflector array (magenta) that is mounted in the field free region after the stack. The deflector consists of 16 electrodes (equal segments of a cylinder) that capable of optimizing the position of the ion cloud on the detector, this was unused in this work and all plates were grounded.

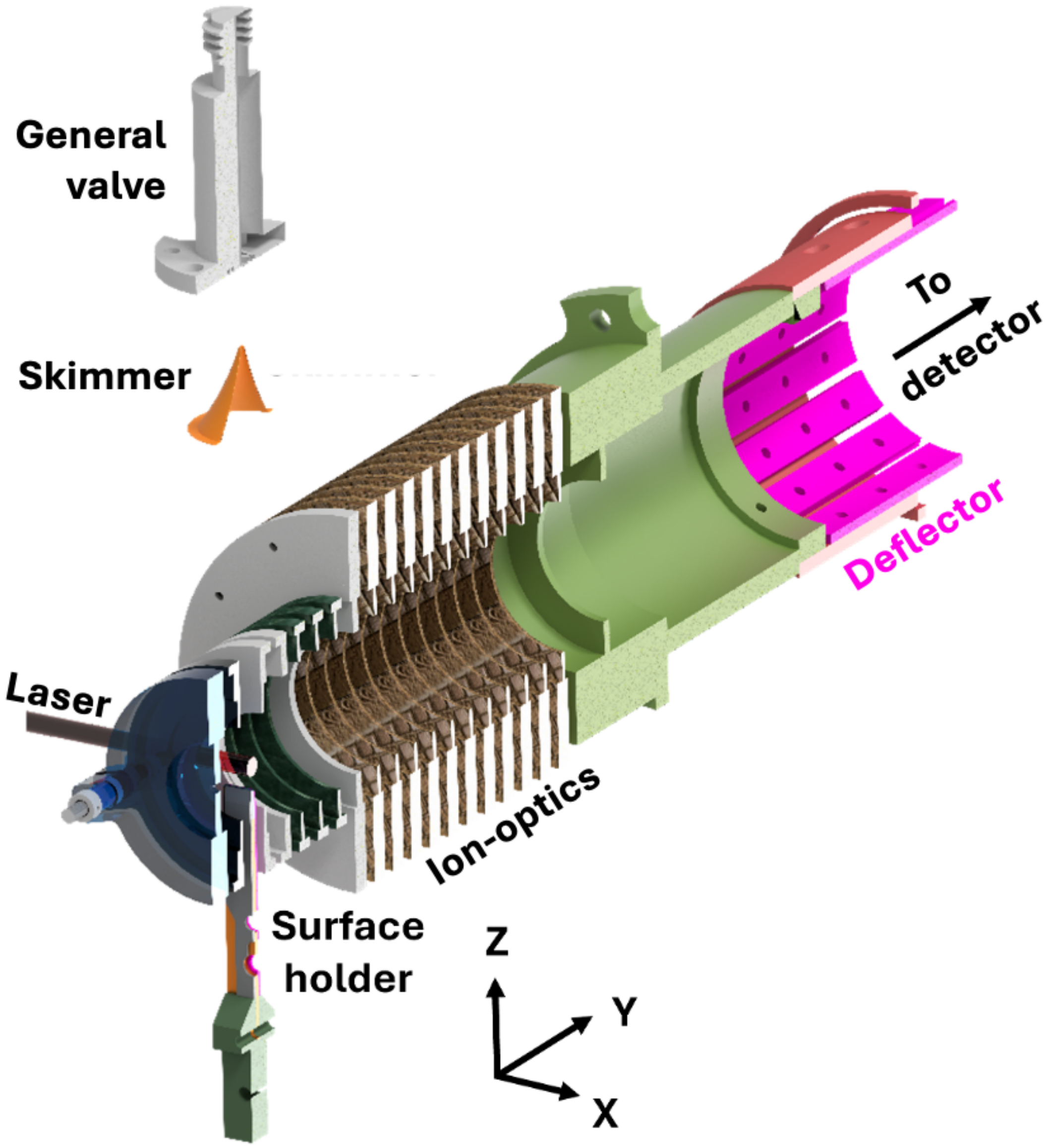

Figure S3. *Half section view of the full ion-optics stack including surface holder, laser, valve and skimmer, see text for details. N.B. this is an expanded version of Fig. 2 of the main text showing additional details of the ion-optics, deflector, and the valve and skimmer that produce the surface normal molecular beam.*

SI-2.2 HOPG surface

The HOPG surface used (AGF1800-1-1 from Agar Scientific) was ZYA grade with a mosaic spread of 0.4°± 0.1° and virtually no steps seen on the cleaved faces. HOPG specimens are layered polycrystals resembling a mosaic of microscopic monocrystal grains that are slightly disoriented with respect to each other, the mosaic spread quantifies the amount of disorientation.

SI-2.3 Molecular Beam Timing

As reported in the main paper we are using a General Valve (series 9) to produce our molecular beams, these valves produce long gas pulses with the fastest and coolest molecules at the start of the pulse. For the experiments reported the valve-laser delay is set to the peak of the molecular beam pulse (as shown in Figure S4 below), this allows time for this early well-defined part of the gas pulse to collide with the surface and for scattered molecules with a large range of speeds to return to the ionization region. The delay of 150 μs from the very start of the gas pulse (80 μs from the 10th centile) is sufficient to allow molecules to collide with the surface and return even if their speeds are as low as 100 m/s at all L-S distances. The incoming times are calculated using a molecular beam speed of 1780 m/s, and return times are calculated for a range of speeds that were detected in the experiments. See Table S2 below for details.

SI Table S2 Times for molecules to travel from the location of the laser to the surface (ingoing, traveling at molecular beam speed) and from the surface to the laser (outgoing) at different speeds, see text for details.

		Times for outgoing out-going speeds / μs						
L-S distance / mm	***Ingoing times / μs***	***1500 m/s***	***1000 m/s***	***500 m/s***	***400 m/s***	***300 m/s***	***200 m/s***	***100 m/s***
3	*1.7*	*2.0*	*3.0*	*6.0*	*7.5*	*10.0*	*15.0*	*30.0*
5	*2.8*	*3.3*	*5.0*	*10.0*	*12.5*	*16.7*	*25.0*	*50.0*
10	*5.6*	*6.7*	*10.0*	*20.0*	*25.0*	*33.3*	*50.0*	*100.0*

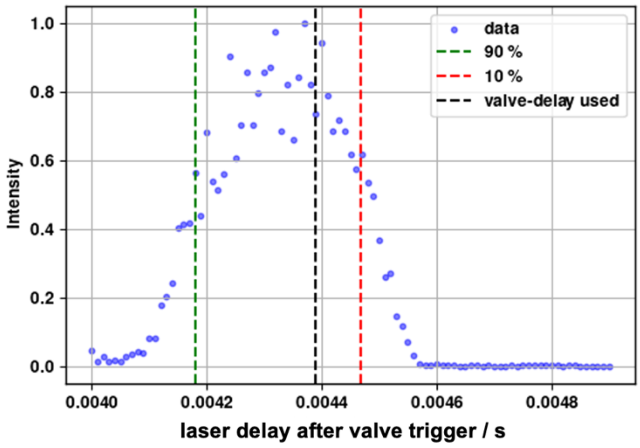

Figure S4 Molecular beam temporal profile showing the laser-valve delay timing used (black dashed line) as well as the 10th (red dashed line) and 90th (green dashed line) percentiles of the integrated molecular beam signal.

SI-3. SIMION Simulations

As described in the main text we upgraded the design of ion optics reported earlier[1] for introducing the surface within the ion optics, with this new design the L-S distance (measured along the Z-axis) can now be decreased from 10 mm to 0 mm, although in the present experiments a minimum of 3 mm was used. To make sure the velocity and spatially map imaging conditions are preserved we carried out 3D SIMION simulations of the experimental apparatus.

The simulations used SIMION's SL Tools to import CAD files of the metal parts of the apparatus as .PA files. Due to the proximity of the sharp stabilizing electrodes to the ionization region it was necessary to use a large number of grid units per mm (gu/mm) to simulate the system satisfactorily: Y-axis (ToF) used 10 gu/mm, and the X and Z-axes used 5 gu/mm. This was the maximum size of simulation possible due to computer memory constraints, the simulations required more than 120GB of RAM.

SI-3.1 Velocity Map Imaging

The voltages required to optimize velocity mapping were calculated using SIMION and are shown in Table S2 below. The list in the table only includes those electrodes whose voltage were controlled by the high voltage power supply. The remaining ion-optics, from the Lens electrode to the final grounded electrode were connected by 220kΩ resistors with identical resistance (within measurable accuracy) to provide an even voltage drop 70 V per electrode.

Table S3 Controlled electrode voltages for VMI. The highest voltage electrode by convention is called the repeller. The remaining electrodes voltages were controlled by resistors, see text for details.

Electrode number	Electrode name	Voltage / V
1	Repeller	1000.00
2		975.14
3		937.86
4	Extractor	913.00
5		894.75
6		876.5
7		858.25
8	Lens	840.00

The ion-optics were mounted on three stainless steel rods positioned so that they would not interfere with the path of the ionization laser, or the paths of the three possible molecular beams. This, along with the presence of the semicylinder of the baffle just above the ion-optics, caused a slight asymmetry in the electric fields in the outer parts of the electrodes as shown in panel (a) of Figure S4. This panel shows the SIMION calculated electric field contours in the scattering plane for a L-S distance of 3 mm. The design of the electrodes minimizes the field asymmetry in the ionization region. Panel (b) in Figure S4 shows the electric field contours perpendicular to those in panel (a), again using 1 V contours to show how the stabilizing electrodes either side of the surface minimize the disruption of the imaging fields.

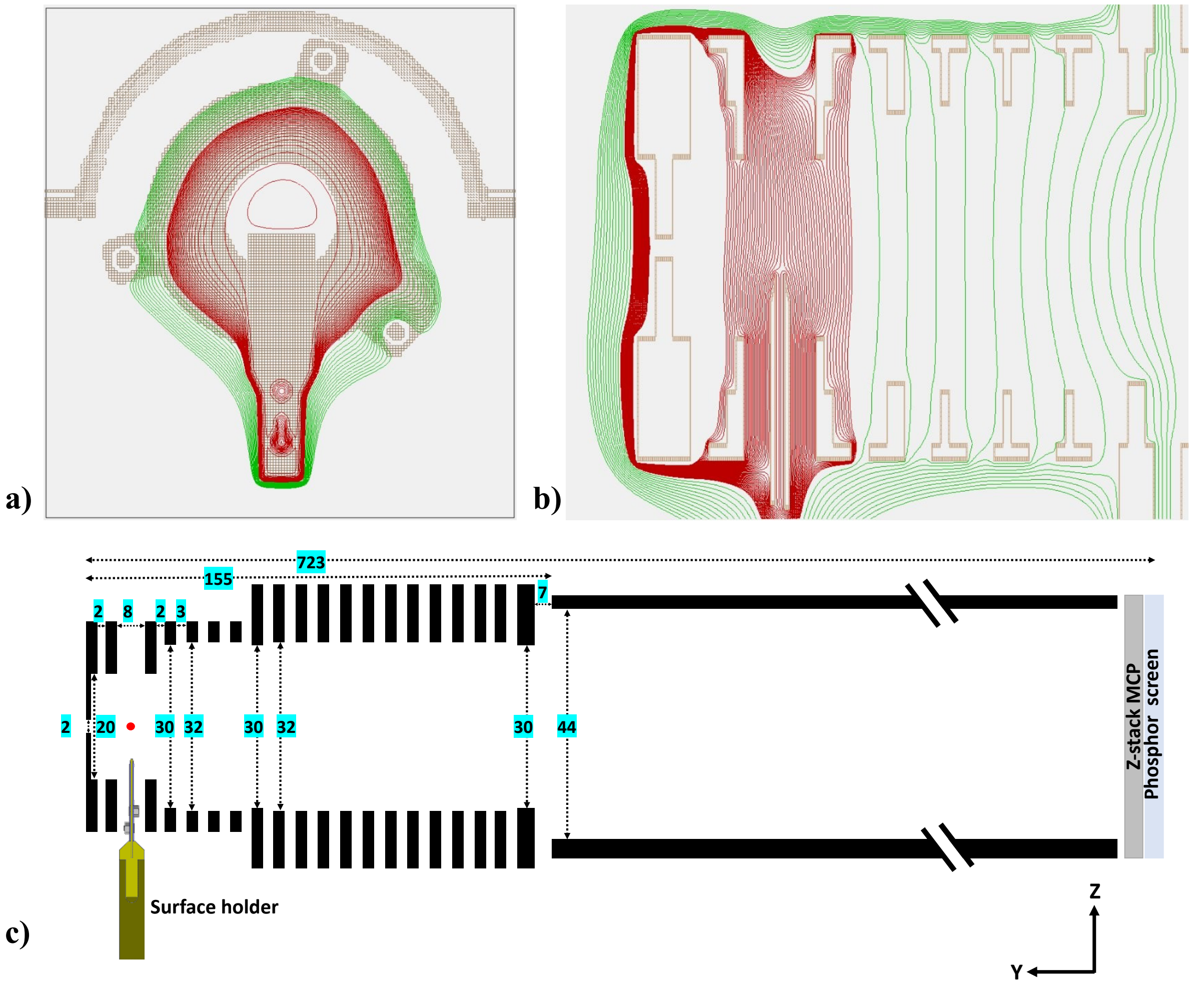

Figure S5. *SIMION calculated electric field contours and Ion optics design. a) Electric field contours in the scattering (XZ) plane (red contours 1 V separation, green contours 12.5 V separation) for L-S = 3 mm. The brown grids show the shape of the metal components; the semicylinder baffle at the top of the image, an annular electrode with three mounting tabs in the center, and one of the surface stabilizing electrodes as the rectangular shape in front of the contours. b) Electric field contours in the ToF plane (YZ), same color scheme and L-S distance as panel (a). c) Schematic of the ion-optics in the YZ-plane with key dimensions of the ion-optics in mm. The ionization region is shown by a red circle above the surface holder.*

SIMION simulations were used to test the velocity mapping region in the 3D model. Ions were given starting positions along the path of the ionization region (limited by the size of the aperture in the ion-optics; ±10 mm) with 0.5 mm increments. At each position along this path ions were generated with an initial direction defined by angles in the scattering plane of ±90° in 5° increments. For each position and initial angle the ions simulated had four different speeds in the scattering plane (403, 806, 1209, and 1612 ms^{-1}) chosen to be representative of the possible velocities in a surface scattering experiment where the incoming molecular beam speed was ~1780 ms^{-1}. The positions that these ions struck the detector were used to calculate

the radius of 'ring' that had been simulated for each speed, as well as the effective scattering angle of the ion. By comparing the radius at ion start positions away from the center with those created by ions started at the center of the ion-optics a radius change could be calculated. Any change in radius illustrates a speed blur, and any change in scattering angle relative to the initial angle of the ion represents a blur in this dimension. Figure S6 shows a plot of the radius change vs start position of ions with four different speeds.

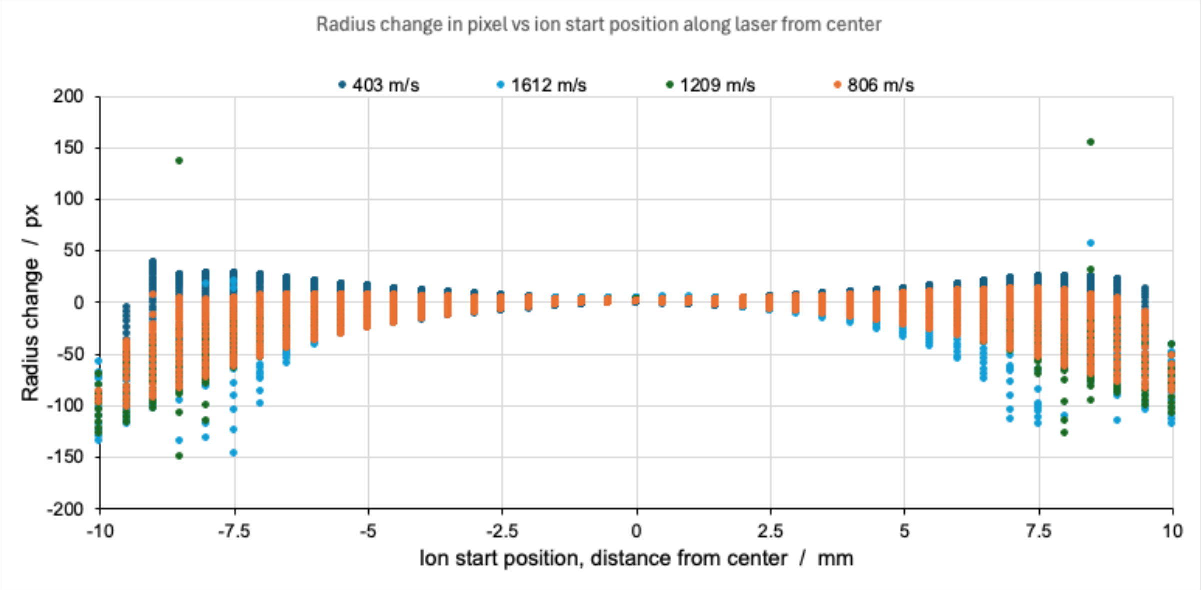

Figure S6. *Results of SIMION simulations; change in radius for arcs of ions started at different positions along the ionization axis.*

For all initial speeds and angles moving the ion creation point away from the center of the experiment caused the velocity mapping to become worse in both speed and angular fidelity. This loss in velocity resolution is small for small displacements from the center but increases for larger displacements. Outside of start positions ±7 mm from the center ion trajectories became more chaotic, especially for higher speeds, as their trajectories took ions close to the inside edges of the ion-optics. For ions with the highest speeds at start positions outside ±9 mm many ions collided with the electrodes themselves and did not reach the detector.

As discussed in the main text literature results from other surface scattering experiments observed broad speed features of several hundreds of ms^{-1}, allowing an acceptance of some speed blurring. A compromise of a speed broadening of up to ~200 ms^{-1} was chosen giving a velocity mapping region of ±5 mm. This is also the length of our surface along the X-axis, beyond which the effect of the stabilizing electrodes would diminish.

SI-3.2 Spatial Map Imaging

By adjusting the voltages on the ion-optics it is also possible to conduct spatial map imaging (SMI) experiments where the position of the ion strike on the detector is proportional to the

position (not the velocity) of the ion when it was created. For the ion-optics used in these experiments SIMION was used to model the electrode voltages for SMI as shown in Table S3. The modeling revealed that the spatial map images were a magnification of the initial positions of ions generated by a factor of 4.01. As the 40 mm diameter detector has a diameter of 1108 pixels this allowed the calculation of a pixel to mm conversion factor of 0.0089972 mm/pixel.

Table S4 Electrode voltages for SMI. The remaining electrodes from the Lens are all connected by high ohm resistors to provide an even voltage drop of 70 V per electrode down to the final grounded (0 V) electrode.

Electrode number	Electrode name	Voltage / V
1	Repeller	1000.00
2		986.38
3		981.43
4	Extractor	974.00
5		952.75
6		931.50
7		910.25
8	Lens	889.00

Figure S7 below shows the result of an SMI experiment, with the first panel showing a thin stripe of NO ions created by the gently focused laser beam intersecting the normal incidence MB. Summing the intensity of the vertical pixels that make up this laser stripe provides the distribution of NO molecules in the molecular beam shown in the 2nd panel of the figure. This distribution was fitted with a gaussian function (reasonable for the expected distribution of molecules in a molecular beam) with a FWHM of 445.88 pixels, which is equivalent to a real space dimension of 4.011 mm.

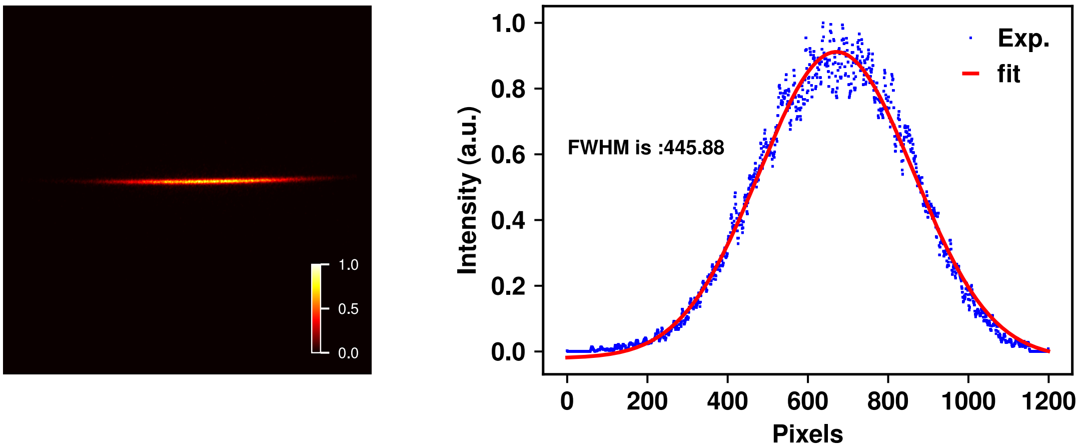

***Figure S7**. Lefthand panel: A spatial Map Image of NO^+ ions in the normal incidence molecular beam. Righthand panel: Intensity along the laser propagation axis (blue squares) fitted with a gaussian function (red line) with a FWHM of 445 pixels.*

SI-3.3 Calibration of VMI

To calibrate the velocity map imaging detector, as mentioned in the main text, the general valve was mounted coaxial to the ion optics along the ToF (Y-axis). For these experiments no surface was present within the ion optics. The velocity mapped ion image resulting from O_2 photodissociation and subsequent ionization at 224.999 nm is presented in the left inset of Figure S8. The image was acquired by dc-slicing the O^+ ions Newton sphere and selecting the center slice by applying a gate width of 25 ns to the MCP detector. The concentric rings in the image correspond to the speeds of the O^+ ions, measured in pixels relative to the center of the image, which is defined as the zero-laboratory-frame speed. Each ring is derived from a specific dissociation pathway, further details on the various channels can be found in the literature.[2,3] The radial intensity of the image is plotted as in the right inset of Figure S8. The main plot in the figure shows the linear relationship of peak positions of the three rings and their known speeds, allowing a velocity calibration to be obtained; For O^+ (m/z = 16 amu), this was 5.4 ± 0.01 ms^{-1} $pixel^{-1}$.

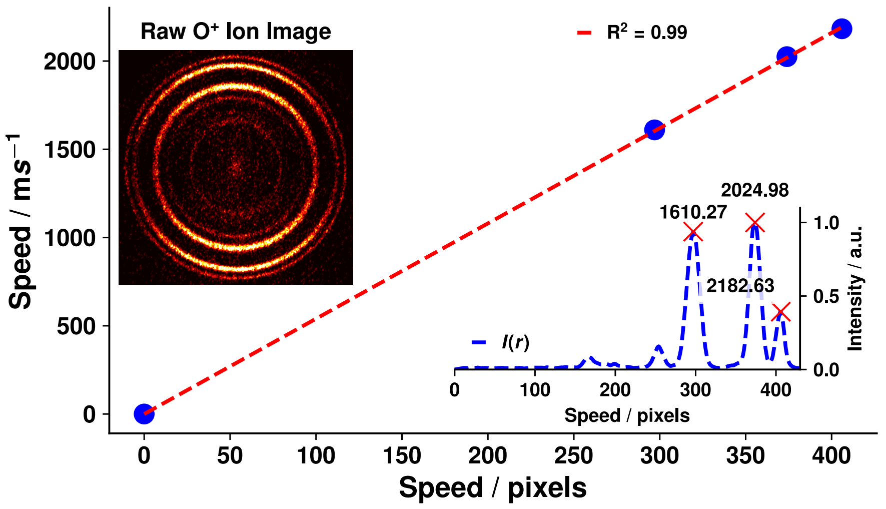

Figure S8. Main plot: Pixel to speed calibration from O_2 photodissociation experiments, data points as blue dots, dashed red line for the linear fit. The left inset shows raw velocity mapped ion image of resultant O^+ ions. The right inset shows radially integrated intensity from raw image as a function of speed in pixel (blue dashed line) with known speeds, in ms^{-1}, labelled (next to red crosses).

Further O_2 photodissociation experiments were carried out to examine the effect of introducing a surface into the ion-optics. Four different surface materials PTFE, HOPG, MICA, and PEEK (each with a breadth of 1 mm) were used to determine the extent to which the surface dielectric perturbs the electric fields. These surfaces were always placed between the two stabilizing electrodes whose potential was adjusted to match the local electric field gradient. O_2 photodissociation experiments were carried out at L-S distances of 3, 5, and 10 mm, as well as without the surface present. In Figure S9, the first four columns show images of O^+ ions acquired with the 1 mm surfaces, while the last column corresponds to a 2 mm surface breadth. The rows indicate the location of the surface.

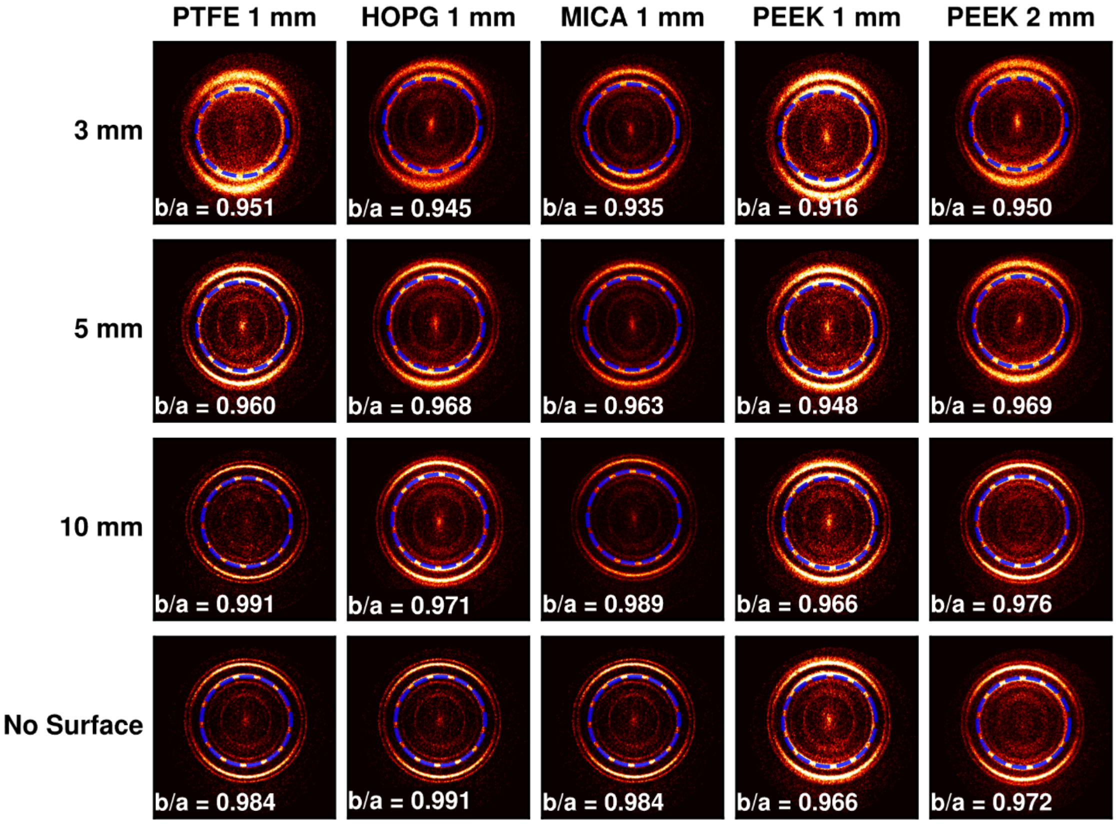

Figure S9. Effect of laser-surface distance and dielectric surface type and thickness on velocity map image quality of O_2 photodissociation images. The top three rows show images for L-S distances of 3, 5, and 10 mm and the bottom row shows images without any surface present in the ion-optics. From left to right the columns show images taken with the four different surface materials (PTFE, HOPG, MICA, PEEK, all 1 mm wide) present, and the rightmost column shows images taken with a 2 mm wide PEEK surface present. All images show a fitted ellipse (blue dashed line) and the calculated b/a ratio for that ellipse in white. See text for details.

As described in the main text, the third outermost ring in each photodissociation image was fitted with an ellipse to quantify the distortion caused by the introduction of the surface. For a perfect circle, the eccentricity is zero, which implies that the minor to major axis (b/a) ratio should be one. The fitted b/a ratio is indicated on each image in Figure S9. As can be seen, aside from minor variations, all ratios remain close to one, indicating that the cylindrical symmetry is largely preserved even at an L–S distance of 3 mm. This behavior is also observed for the PEEK surface with a breadth of 2 mm; however, these images clearly show that the speed resolution deteriorates when compared to those with a breadth of 1 mm. Therefore, a surface of 1 mm breadth was chosen for the scattering experiments.

Introducing any material within ion-optics is expected to distort the electric field, thereby distorting the velocity mapping fields. By using stabilizing electrodes on either side of the surface and minimizing the breadth of the surface the experiment is able to mitigate this effect. The dielectric constants of these surfaces cover a large range of between approximately 2 to

20, demonstrating the capability of the stabilizing electrodes to maintain velocity mapping conditions in the experiment.

SI-3.4 Calibration of VMI from NO_2 for NO species in scattering geometry:

In the scattering geometry the molecular beam assembly is mounted on the surface normal port as shown in Figure S2b and Figure S3. To confirm the quality of the velocity mapping in this geometry, and for speeds similar to those expected in the surface scattering measurements, NO_2 photodissociation experiments were carried out as described in the main text. Due to the NO mass to charge ratio of 30 amu, its arrival time at the detector is expected to longer than that of O^+ ions found in the O_2 photodissociation experiments. Consequently, the speed to pixel calibration differs from that determined in Figure S8.

In these calibration experiments, ~226 nm wavelength light was used to excite the NO_2 molecules to the 2^2B_2 state which subsequently dissociates to produce NO(X) and $O(^1D)$ photofragments. Two subsequent photons ionize the NO(X) fragment via [1+1] REMPI probing the $NO(A^2\Sigma^+ - X^2\Pi)$ (0,0) band, as described in the main text. A 5 % mixture of NO_2 in Ar with 3 bar backing pressure was used to generate a molecular beam, and the detector was gated (20 ns) to record the center slice of the ion cloud. A vertically polarized laser beam was focused with a 30 cm focal length lens to ionize the molecular beam.

Five NO rotational transitions were selectively probed: j = 6.5 and 13.5 from the R_1 branch, and j = 19.5, 26.5, and 33.5 from the P_{21} branch. These transitions were chosen as they are well isolated from any other overlapping lines. Velocity mapped ion images were recorded for each transition (see top row of Figure S10). In these images, the outer ring corresponds to the NO^+ ions produced from NO_2 photodissociation, while the signal intensity near to the center originates from trace amounts of NO in the molecular beam (j = 6.5 and 13.5) or NO produced via photodissociation of N_2O_4 dimers formed in the molecular beam during expansion.

The speed (ms^{-1}) of the NO photofragments for each transition was calculated: v = 937 (R_1(6.5)), 909 (R_1(13.5)), 855 (P_1(19.5)), 777 (P_1(26.5)), and 664 (P_1(33.5)).[4] Using these known velocities and the peak position of radially summed NO^+ intensity distribution (see middle row of Figure S10), a calibration factor of 3.98 ± 0.01 ms^{-1} $pixel^{-1}$ was obtained from the linear fit shown in the bottom panel of Figure S9. This calibration factor agreed with the mass converted calibration factor obtained from the O_2 photodissociation experiments and was subsequently used to convert detector pixels to molecular speeds in the surface scattering experiments.

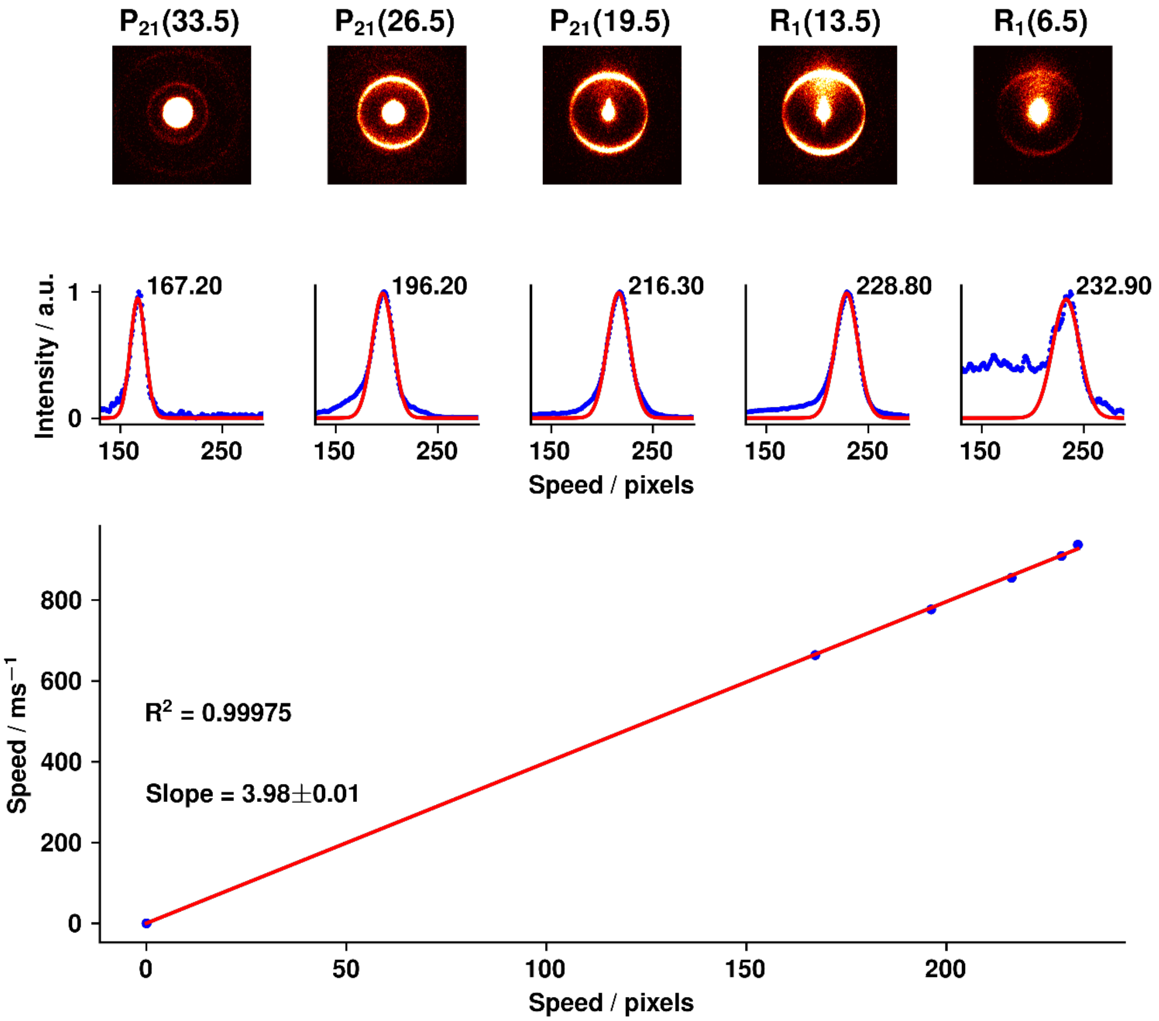

Figure S10. Top row: Raw velocity map images of NO^+ ions formed from photodissociation of NO_2 at ~226 nm, transitions used for NO ionization are shown above each image. Middle row: Speed distributions of NO^+ photofragments in pixels for each transition, radially summed data from images (blue dots) with gaussian fits to the peaks of the rings (red lines). Bottom panel: pixel to velocity calibration with experimental data derived from the speed distributions in the middle row (blue dots) and linear fit (red solid line).

SI-4. ToF of Ions Outside the Mapping Volume

As discussed in the main text; a broad detector gate width was required as NO^+ ions that are created far from the center of the ion-optics experience slightly different electric fields from those at the center of the mapping region. This increases their flight time to the detector, decoupling time-of-flight from only being due to initial out of plane scattering. This well-known phenomenon is one of the parameters that must be considered when VMI-ion-optics designs are optimized.[5] This is an important factor to consider if surface-VMI experiments are being run in dc-slicing mode (i.e., gating the detector over a narrow time window of ion packet) as was done for the O_2 and NO_2-photodissociation experiments discussed above. In the case of surface-scattered NO^+, slicing the center of the ion packet will result in missing the signal at large scattering angles due to the relationship between detection point and scattering angle, as demonstrated in Figure S11. This figure shows an image of the center slice of the ion packet compared with one taken at 10 ns longer ToF and clearly shows that later time slices detect only the wider scattering angles. If slices are taken with smaller time steps between them then one can see a continuous progression from the central slice to a series of V shaped scattering images with increasing opening angles as the time from the central slice is increased.

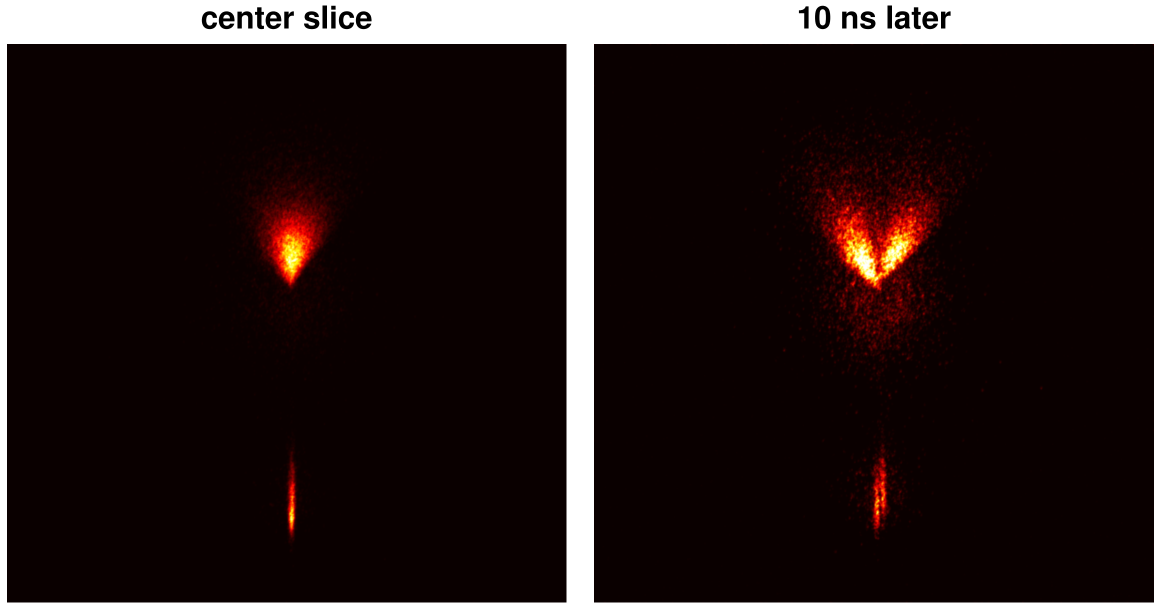

Figure S11. Raw Images taken for NO^+ ions scattering off HOPG at L-S = 5 mm, with dc-slice gate widths of 20 ns. Left panel shows the slice containing the centre of the ion cloud. Right panel shows the ion cloud slice 10 ns later in the ToF. The bifurcation in the right image is attributed to ions that are created further away from the centre of the laser axis and thus experience a slightly different electric field causing their flight time to be delayed.

This effect was also demonstrated in SIMION simulations of the experiment which showed that ions created further from the center of the ionization region will have a slightly longer ToF, as illustrated in Figure S12. The simulations in this figure are for only one product speed, the same effects are also observed for other speeds and increase the relative ToF delay, these have

been omitted for clarity. Because of the experimental geometry only molecules scattered from the surface at large (relative to the surface normal) scattering angles will reach the laser at these outermost points. If this phenomenon was not understood and experiments were carried out using a narrow dc-slice it would lead to the impression that larger scattering angles had an impossible velocity component going away from the detector. Thus NS-VMI experiments must be carried out using crush imaging and optical slicing to ensure that all 'in-plane' scattered molecules are detected by the laser.

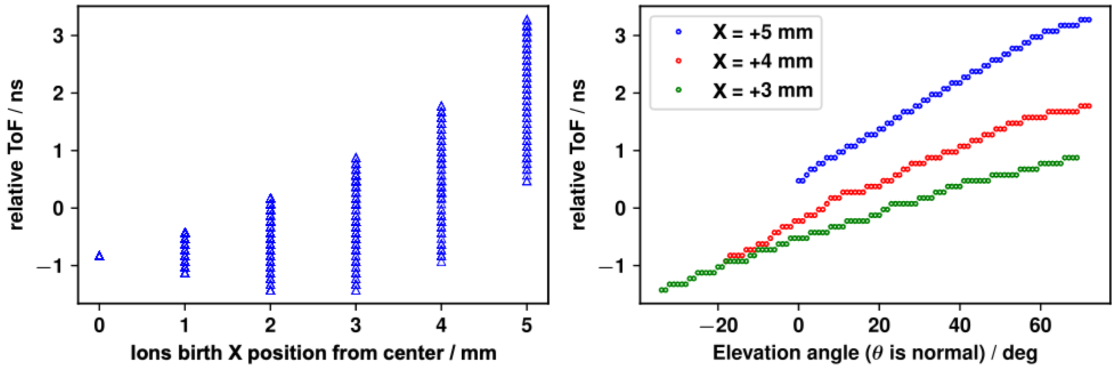

Figure S12. Ion time-of-flight (ToF) analysis of NO^+ ions generated along the laser (X) axis. Left hand panel: ToF as a function of ion creation position relative to the centre along the laser propagation axis, showing the change in flight time caused by ionization location. Right hand panel: ToF as a function of ion elevation (scattering) angle, showing the variation in arrival times due to angular displacement from the central axis.

SI-5. Relative Populations of NO

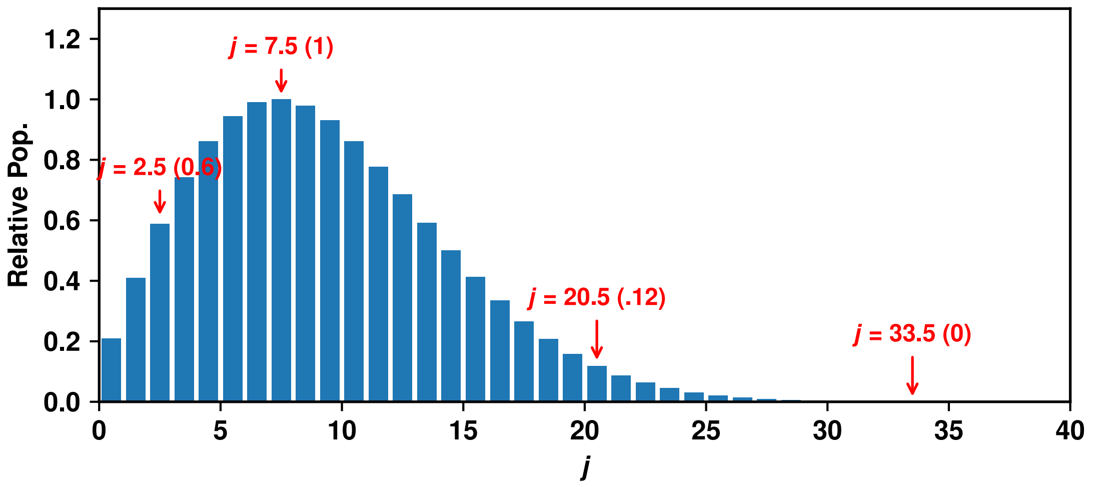

Figure S13. *Relative populations of NO molecules in a thermalized sample at 293 K. The four rotational quantum states measured in this work are labelled in red text, with their relative populations included in brackets. N.B. this data is included in Table 1 in the main text.*

SI-6. Scattering Results

This section contains both figures that are additional to the main text and more complete versions of the figures in the main text, i.e. the figures below will contain the data from *all* quantum states measured at *all* laser-surface distances.

SI-6.1. Background Subtraction

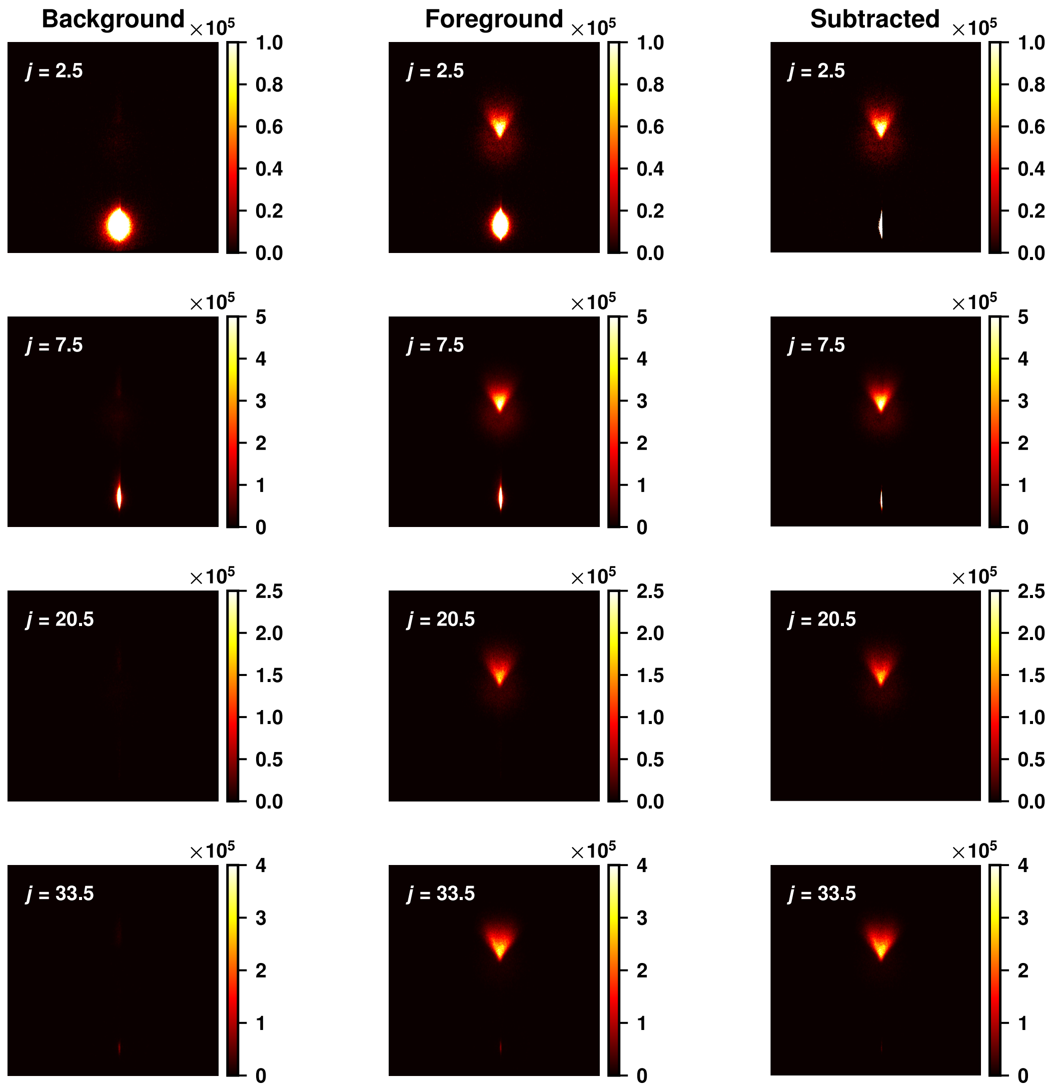

Figure S14. Raw Images of NO^+ ions acquired for four rotational lines j = 2.5, 7.5, and 20.5 of the R_{21} branch and j = 33.5 of the Q_1 branch taken with an L-S distance of 10 mm. The left-hand column shows the background images taken without any surface present in the ion-optics; these images include MB signal towards the bottom of the image for the low j states and at much lower intensity any background signal due to NO molecules that have thermalised due to collisions with the ion-optics and baffle. The middle column shows images taken with the surface present which include the signals present in the first columns as well as the surface scattering signal. The right-hand column shows the results of subtracting the left-hand column from the middle column i.e. the background subtracted images.

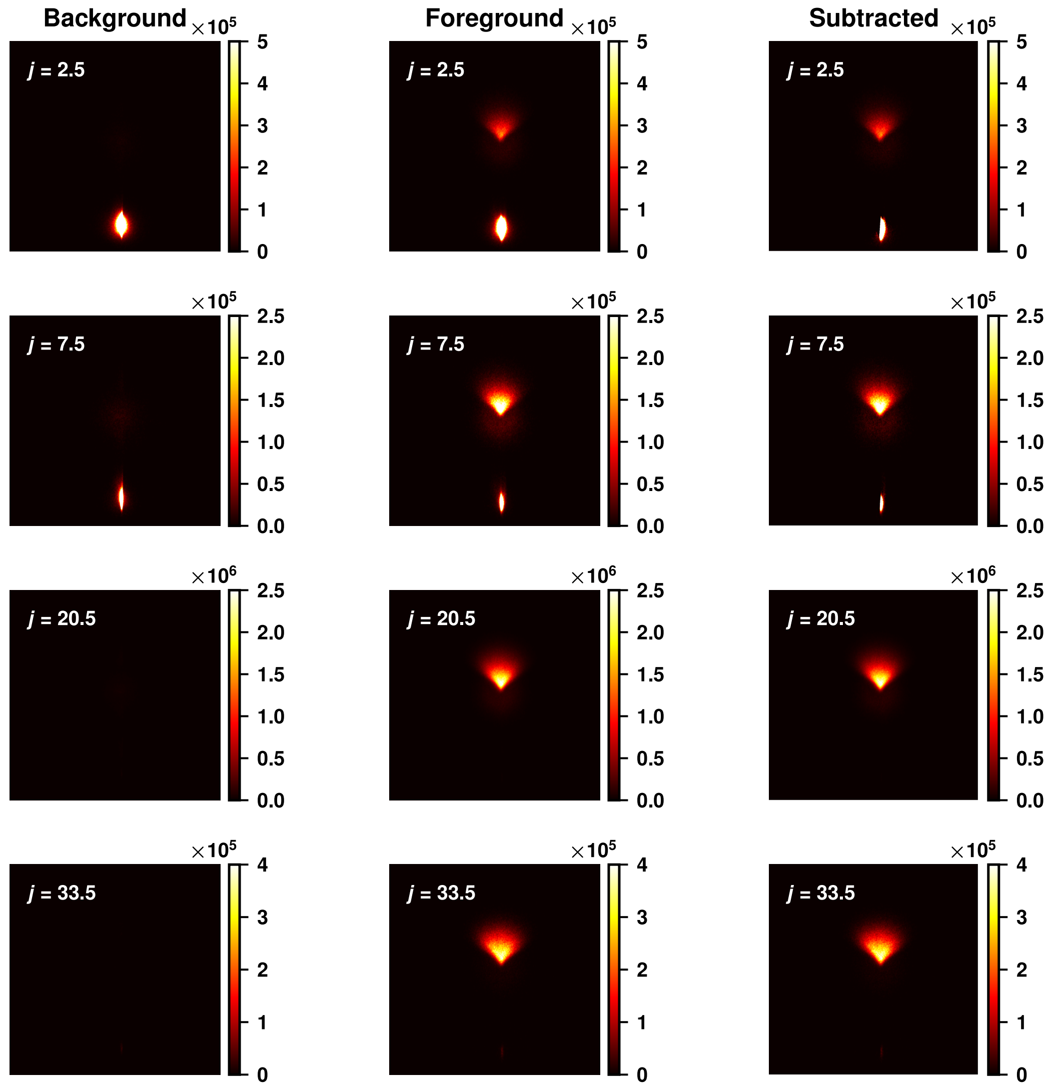

Figure S15. Raw Images of NO^+ ions acquired for four rotational lines *j* = 2.5, 7.5, and 20.5 of the R_{21} branch and *j* = 33.5 of the Q_1 branch taken with an L-S distance of 5 mm. The left-hand column shows the background images taken without any surface present in the ion-optics; these images include MB signal towards the bottom of the image for the low *j* states and at much lower intensity any background signal due to NO molecules that have thermalised due to collisions with the ion-optics and baffle. The middle column shows images taken with the surface present which include the signals present in the first columns as well as the surface scattering signal. The right-hand column shows the results of subtracting the left-hand column from the middle column i.e. the background subtracted images.

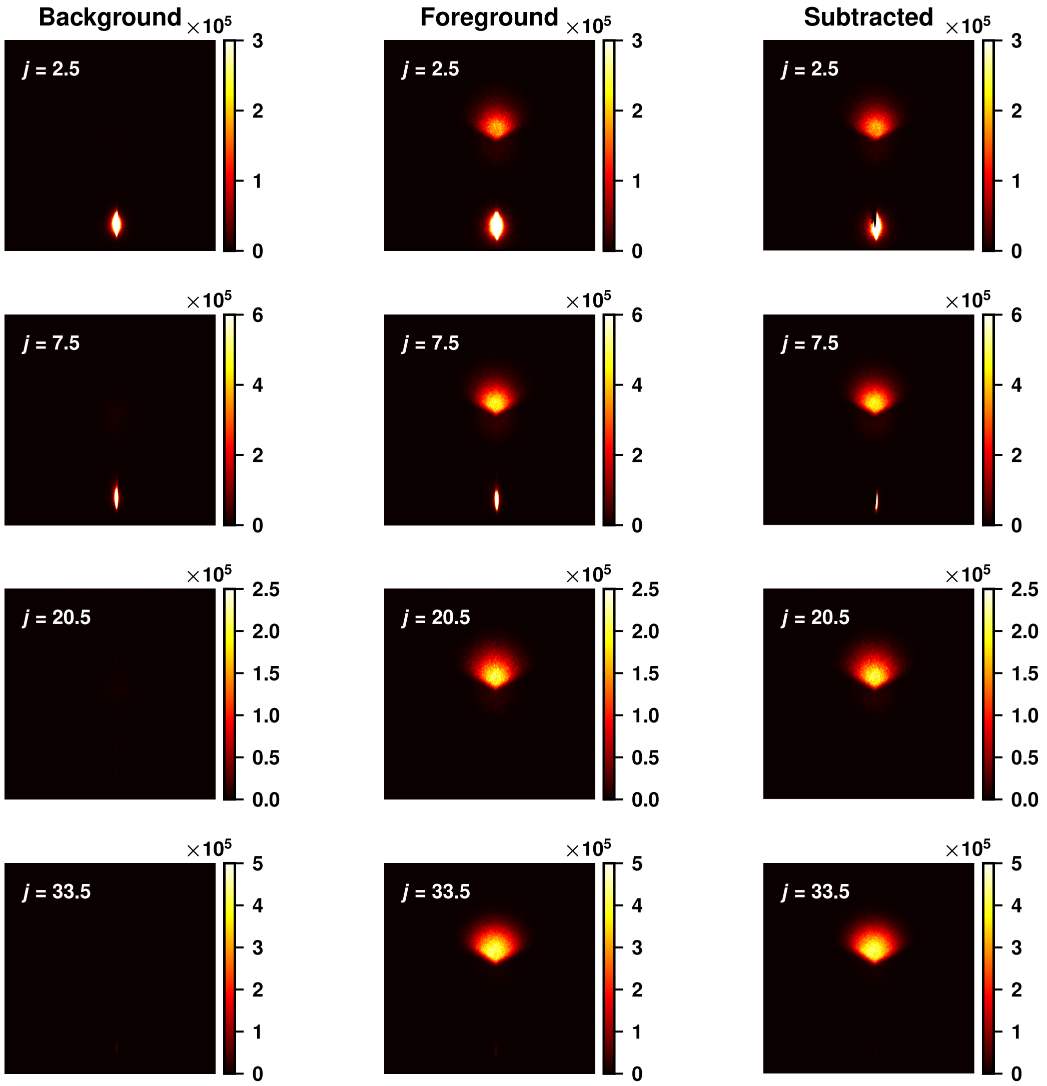

Figure S16. Raw Images of NO^+ ions acquired for four rotational lines *j* = 2.5, 7.5, and 20.5 of the R_{21} branch and *j* = 33.5 of the Q_1 branch taken with an L-S distance of 3 mm. The left-hand column shows the background images taken without any surface present in the ion-optics; these images include MB signal towards the bottom of the image for the low *j* states and at much lower intensity any background signal due to NO molecules that have thermalised due to collisions with the ion-optics and baffle. The middle column shows images taken with the surface present which include the signals present in the first columns as well as the surface scattering signal. The right-hand column shows the results of subtracting the left-hand column from the middle column i.e. the background subtracted images.

SI-6.2. Background Subtracted Scattering Images

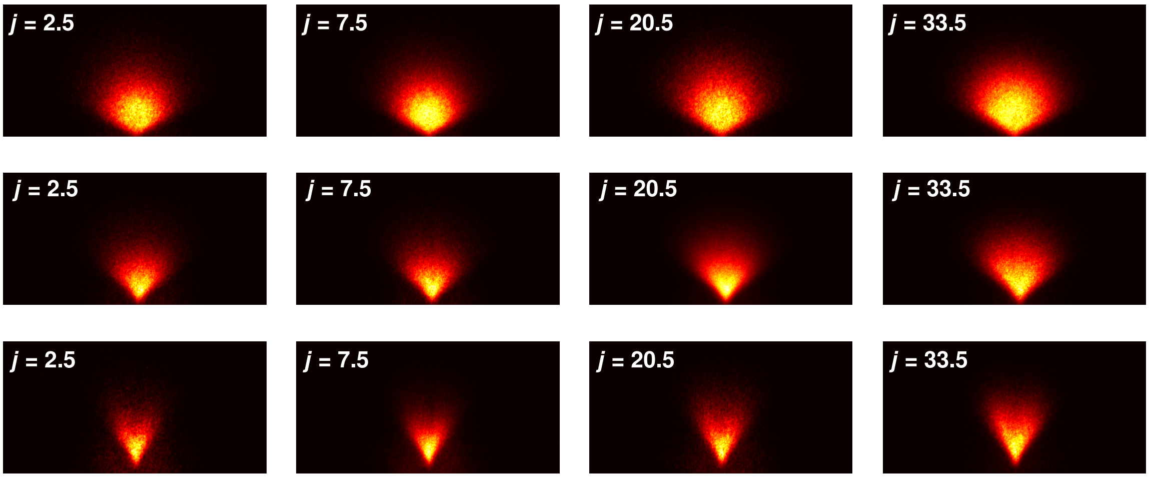

Figure S17. Raw NO^+ ion images of the surface scattered (SS) region after subtraction of background, acquired for four rotational lines j = 2.5, 7.5, and 20.5 of the R_{21} branch and j = 33.5 of the Q_1 branch at L-S distances of 10 mm (bottom row), 5 mm (middle row), and 3 mm (top row). N.B. this is a version of Figure 5 in the main text showing all measured states at all L-S distances, see main text for details.

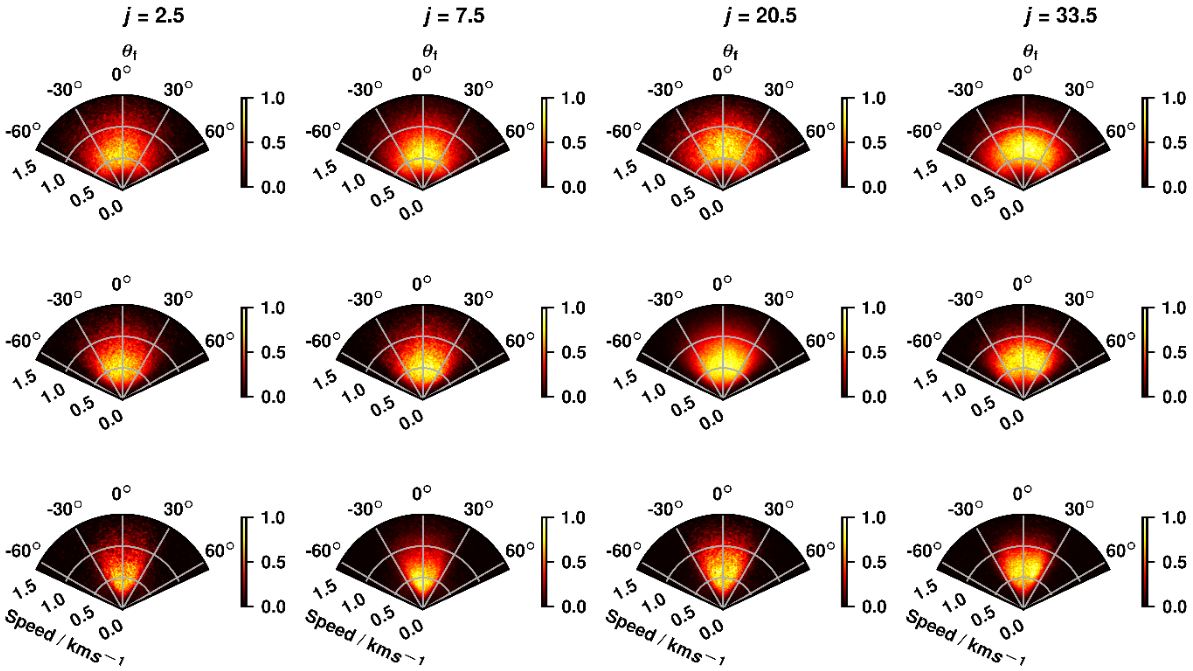

Figure S18. Density-flux-corrected intensity map of NO^+ ions for j = 2.5, 7.5, 20.5, and 33.5 (left to right) and for L-S distance 10, 5, and 3 mm (bottom to top). N.B. this is a version of Figure 6 in the main text showing all measured states at all L-S distances, see main text for details.

SI-6.3. Speed and Angular Distributions

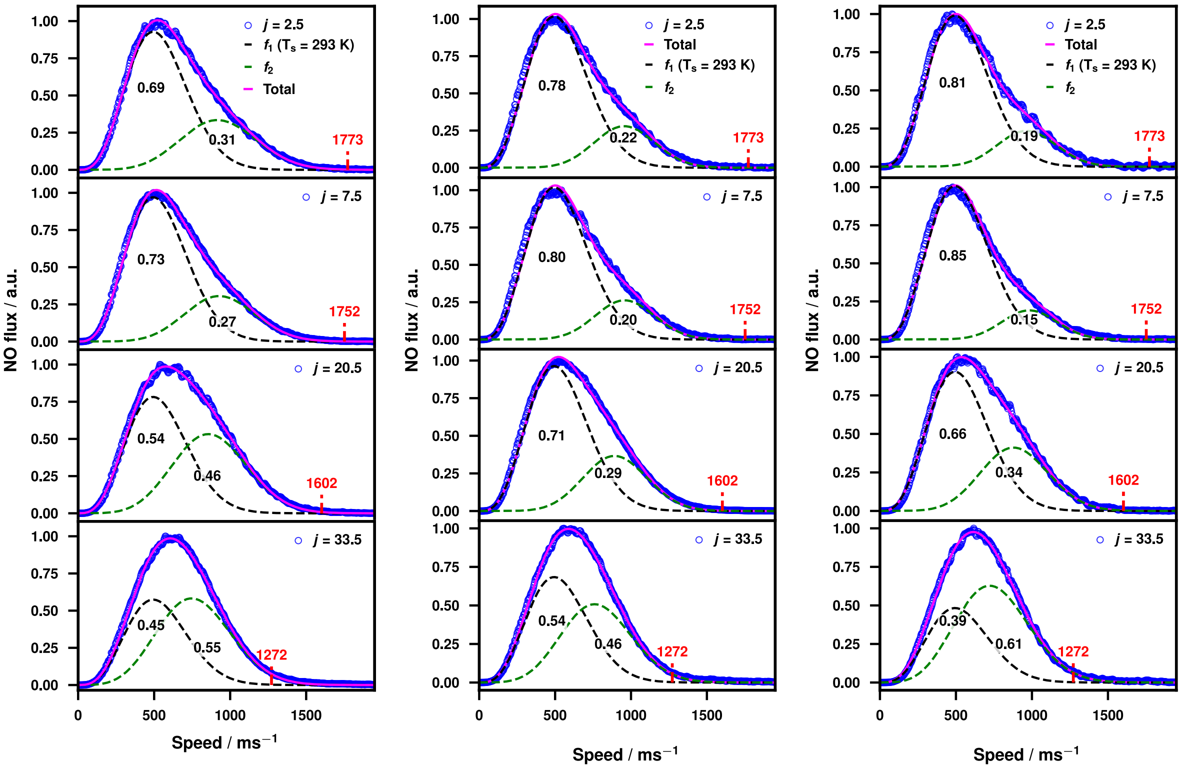

SI Figure19. Angularly integrated distributions of flux as a function of speed for all recorded quantum states for L-S distances of 3, 5, and 10 mm (left to right). Each panel shows the experimental data as blue circles with a two-component fit to the data shown as a magenta line. The surface temperature related f_1 component of the fit is shown as a black dashed line, and the additional component is the green dashed line, the relative areas of the two components are shown in text boxes under each curve (see main text for details). The speed cutoff, as calculated using the mean MB speed & energetics of the scattering, is shown as a labelled dashed vertical red line (see main text for more details). N.B. This is a version of Figure 7 in the main text showing all measured states and L-S distances, see main text for details.

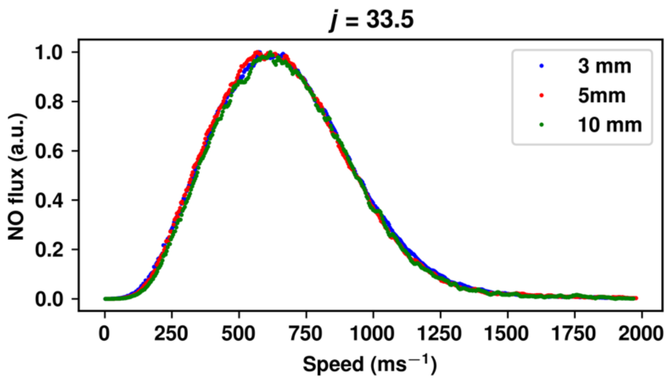

Figure S20. *Comparison of angularly integrated distributions of flux as a function of speed for the j = 33.5 state at three L-S distances. The plots shows that the speed distributions measured are not affected by the change in L-S distance.*

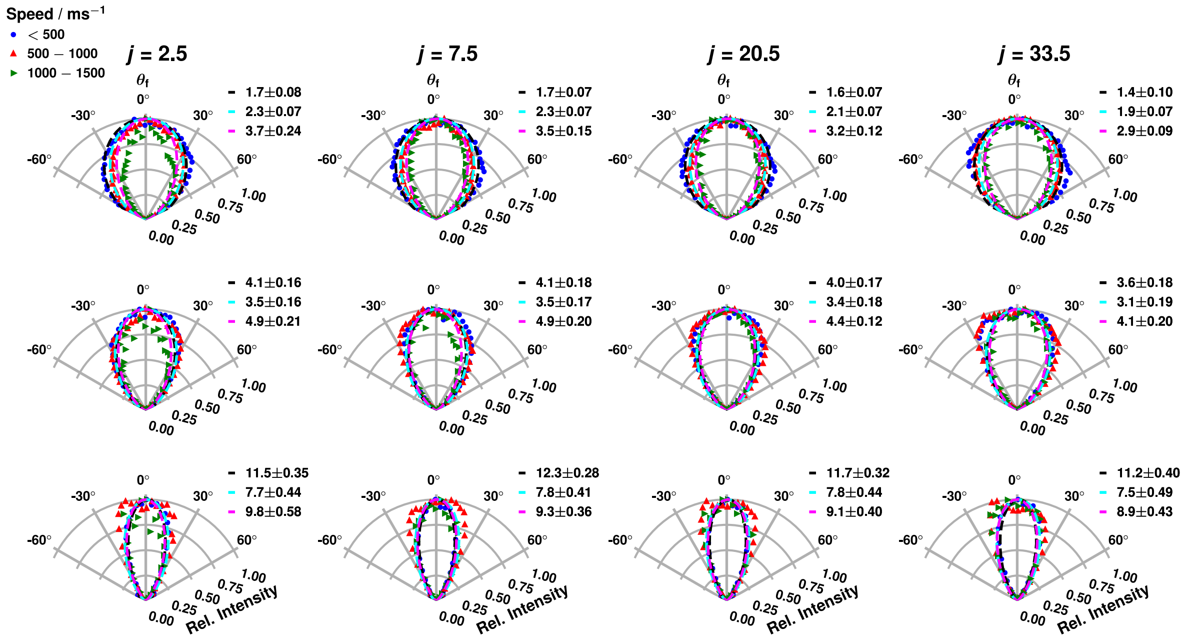

Figure S21. Experimental angular distributions with cos^{n}θ fits, recorded at each L-S distance (bottom row: L-S = 10 mm, middle L-S = 5 mm, top L-S = 3 mm). The fitted *n* values of the *cosine^n* distributions are shown with their errors next to each plot. The plots show the angular distributions for each of three speed regions: speeds below 500 ms^{-1} (blue circles); speeds between 500-1000 ms^{-1} (red upwards triangles); speeds between 1000-1500 ms^{-1} (green sideways triangles). N.B. This is a version of Figure 8 in the main text showing all measured states and L-S distances for fits to the full angular range of data, see main text for details.

REFERENCES

(1) Hadden, D. J.; Messider, T. M.; Leng, J. G.; Greaves, S. J. Note: Velocity Map Imaging the Scattering Plane of Gas Surface Collisions. *Review of Scientific Instruments* **2016**, *87* (10), 1–4. https://doi.org/10.1063/1.4965970.

(2) Sun, Z.; Scheidsbach, R. J. A.; Banerjee, A.; Eppink, A. T. J. B.; Wei, Y.; Hua, Z.; Yang, X.; Qin, Z.; Xu, X.; Zheng, X.; Farooq, Z.; Chen, Z.; Parker, D. H. Multiphoton Dissociation Dynamics of Molecular Oxygen O2 via Two-Photon Resonant Rydberg States in the UV Region. *Journal of Chemical Physics* **2025**, *162* (5). https://doi.org/10.1063/5.0251055.

(3) Parker, D. H.; Eppink, A. T. J. B. Photoelectron and Photofragment Velocity Map Imaging of State-Selected Molecular Oxygen Dissociation/Ionization Dynamics. *J Chem Phys* **1997**, *107* (7), 2357–2362. https://doi.org/10.1063/1.474624.

(4) Luxford, T. F. M. The Stereodynamics of the Inelastic Collisions of NO(A2Σ+) with Atoms and Molecules, Heriot-Watt University, Edinburgh, 2017.

(5) Plomp, V.; Gao, Z.; van de Meerakker, S. Y. T. A Velocity Map Imaging Apparatus Optimised for High-Resolution Crossed Molecular Beam Experiments. *Mol Phys* **2021**, *119* (1–2). https://doi.org/10.1080/00268976.2020.1814437.
